# Decomposition of spontaneous brain activity into distinct fMRI co-activation patterns

**DOI:** 10.3389/fnsys.2013.00101

**Published:** 2013-12-04

**Authors:** Xiao Liu, Catie Chang, Jeff H. Duyn

**Affiliations:** Advanced MRI Section, Laboratory of Functional and Molecular Imaging, National Institute of Neurological Disorders and Stroke, National Institutes of HealthBethesda, MD, USA

**Keywords:** resting-state network, non-stationary connectivity, network dynamics, clustering, dynamic connectivity

## Abstract

Recent fMRI studies have shown that analysis of the human brain's spontaneous activity may provide a powerful approach to reveal its functional organization. Dedicated methods have been proposed to investigate co-variation of signals from different brain regions, with the goal of revealing neuronal networks (NNs) that may serve specialized functions. However, these analysis methods generally do not take into account a potential non-stationary (variable) interaction between brain regions, and as a result have limited effectiveness. To address this, we propose a novel analysis method that uses clustering analysis to sort and selectively average fMRI activity time frames to produce a set of co-activation patterns. Compared to the established networks extracted with conventional analysis methods, these co-activation patterns demonstrate novel network features with apparent relevance to the brain's functional organization.

## Introduction

A growing body of neuroimaging research is reporting on the phenomenon of spontaneous neural activity occurring during rest, in the absence of overt behavior (Biswal et al., 1995; Arieli et al., 1996; Leopold and Maier, 2012). Functional magnetic resonance imaging (fMRI) (Ogawa et al., 1992) studies of the nature of this activity have revealed multiple spatial patterns of temporally correlated signal fluctuation that cover much of the brain, and often align with the established systems that support specialized brain functions (Biswal et al., 1995; Cordes et al., 2000; Hampson et al., 2002; Greicius et al., 2003; Fox et al., 2006). Based on this, it has been hypothesized that these correlational patterns of fMRI activity (often called “resting-state networks,” or RSNs) (Fox and Raichle, 2007) indirectly result from spontaneous electrical activity in a number of distinct, large scale, and function-specific neuronal networks (NNs). Interpretation of RSNs in terms of the NNs supporting the major brain functions is an important goal of current neuroimaging research (Fox and Raichle, 2007; Biswal et al., 2010; Zhang and Raichle, 2010).

To tackle this challenging problem, a number of methods have been applied to analyze resting-state fMRI signals, including “seed”-based correlation analysis (Biswal et al., 1995; Fox et al., 2006), clustering based on temporal characteristics (Cordes et al., 2002; Mezer et al., 2009) and spatial or temporal independent component analysis (ICA) (Kiviniemi et al., 2003; Beckmann et al., 2005; Smith et al., 2012). These methods generally make implicit or explicit assumptions about the source signals underlying spontaneous fMRI activity, including stationarity of inter-regional interactions, and/or a statistical independence. The extent to which these assumptions reflect the nature of NNs determines how accurately they are represented by RSNs.

Recent studies have provided evidence that spontaneous brain activity may be non-stationary (Chang and Glover, 2010; Allen et al., 2012; Hutchison et al., 2012; Rack-Gomer and Liu, 2012), and in fact may be dominated by brief instances of spontaneous co-activation of brain regions (Tagliazucchi et al., 2012; Liu and Duyn, 2013; Wu et al., 2013). This has inspired a novel analysis approach that temporally decomposes conventional RSNs into multiple co-activation patterns by selective averaging of single fMRI time frames (Liu and Duyn, 2013). Here we extend this approach and perform a comprehensive analysis of a publicly accessible fMRI database to extract 30 spatial patterns of spontaneous activity (termed co-activation patterns or CAPs) that are biologically plausible and show distinct differences from networks extracted with conventional methods.

## Methods

### Analysis approach

The proposed analysis approach is based on the notion that spontaneous activity may be dominated by brief activations and deactivations involving many (possibly overlapping) brain regions. It differs from point process analysis (PPA), which models activity underlying fMRI signals as point processes and then examines the conditional distribution of these processes given its occurrence at a specific seed region (Tagliazucchi et al., 2012; Wu et al., 2013), in that it is not specifically geared toward detecting neuronal-avalanche type activity. Rather than selectively averaging time points of activity increases in a seed region, the method proposed here classifies and averages time points with similar spatial distributions of activity using the *k*-means clustering algorithm. It thus extends the seed-based approach presented in a previous study (Liu and Duyn, 2013) to a data-driven, whole-brain analysis.

Clustering is a procedure for classifying a set of objects into different groups such that within group differences are smaller than across group differences. An approach popular with fMRI studies is the *k*-means clustering method, which has previously been applied to classify brain voxels based either on their temporal dynamics (Cordes et al., 2002; Mezer et al., 2009) or correlation profiles (Anderson et al., 2010; Kelly et al., 2012). Here, in contrast, we apply *k*-means clustering to classify fMRI time points (fMRI image volumes) based on their *spatial* similarity.

After reformatting fMRI brain volumes into a set of *m*-dimensional vectors {*t*_1_, *t*_2_, …, *t*_*n*_} (where *m* = the number of brain voxels, based on a brain mask created from the 152-brain MNI template), *k*-means clustering is applied to partition the vectors into *k* clusters ***R*** = {*R*_1_, *R*_2_, …, *R*_*k*_} (Gluck and Myers, 1997) such that the sum of within-cluster distances *J* (Equation 1) is minimized:

(1) $$J=\sum_{i=1}^{k}\sum_{t_{j}\in R_{i}}d\text{​}\left(t_{j},\mu_{i}\right)$$

where μ_*i*_ is the mean of fMRI volumes in *R*_*i*_, and *d*(•) represents the distance between two vectors, which we define here to be 1 minus their Pearson's correlation coefficient.

### Dataset

The resting-state fMRI dataset used in this study was obtained from the 1000 Functional Connectomes Project (FCP) (http://www.nitrc.org/projects/fcon_1000/) (Biswal et al., 2010). It includes data from studies performed independently at three different sites (Baltimore, Berlin_Margulies, and Cambridge_Buckner) with approval from their respective ethics committees. Due to computational limitations, we focused our analysis on data from 102 subjects (mean age: 24.4 ± 6.6, range: 18-44; sex: 64 females) selected from all 3 sites. Detailed information regarding each dataset and the major MR acquisition parameters can be found at the FCP website.

### Pre-processing of resting-state fMRI signals

FCP analysis scripts (version 1.1-beta, available at http://www.nitrc.org/frs/shownotes.php?release_id=938) (Biswal et al., 2010), which employs AFNI (Cox, 1996) and FSL (http://www.fmrib.ox.ac.uk/fsl/) (Smith et al., 2004) software packages, were used to pre-process the fMRI signals (with minor modifications, described below). The typical pre-processing steps for the analysis of resting state data were applied, including image coregistration to correct for head motion, spatial smoothing with a Gaussian kernel (FWHM = 4 mm), temporal filtering with a band-pass filter (0.005–0.1 Hz), and the removal of linear and quadratic temporal trends. Additionally, the time series of ROIs in the white matter and cerebrospinal fluid (CSF), 6 affine motion parameters, as well as the brain-averaged (global) signal, were used as nuisance variables to be regressed out from the data. Given that global signal regression (GSR) may introduce artificial anti-correlation between regions (Fox et al., 2009; Murphy et al., 2009; Saad et al., 2012) our analysis was also performed without the GSR step (Figure S1).

The fMRI data was first co-registered to the high-resolution anatomical (T_1_-weighted) images acquired from the same subject and then normalized to the 152-brain Montreal Neurological Institute (MNI) normalized space. Here, as departure from the original FCP scripts, the registration between the functional and anatomical images was implemented using the “align_epi_anat.py” program (Saad et al., 2009) in AFNI, which was found to provide a better registration in the superior-inferior direction. The pre-processed fMRI data were then resampled at the 3 × 3 × 3 mm^3^ resolution of the MNI normalized brain space. Finally, for each voxel, the fMRI signal was temporally normalized by subtracting its mean and then dividing by its temporal standard deviation (*SD*).

### Extraction of CAPs

The clustering was applied to all 13382 fMRI volumes acquired from all 102 participants. After clustering, the fMRI volumes (also referred to as “time frames”) assigned to the same cluster were simply averaged, resulting in *k* maps that we define as CAPs. These CAPs were then normalized by the standard error (within cluster and across fMRI volumes) to generate *Z*-statistic maps, which quantify the degree of significance to which the CAP map values (for each voxel) deviate from zero. We also calculated three quantities for each CAP based on its raw map: (1) the occurrence rate, which was calculated by dividing the number of fMRI time frames belonging to a given CAP by the total number of time frames; (2) the within-cluster similarity, calculated as the average spatial correlation of all within-cluster volumes to their mean; and (3) the polarity, calculated as the sum of the mean of positive map values and that of negative map values, such that the resulting sign indicates whether the CAP is dominated by activation or de-activation.

To suppress the contribution of measurement noise, only the largest signal changes were considered for calculation of distances for the clustering procedure. This was achieved by applying a mask to the fMRI volumes that only admitted the 10% highest and 5% lowest signal values, and discarded regions with less than 6 inter-connected (in 3D) voxels. This masking was only performed for the clustering and not for the within-cluster averaging procedure for calculation of the grand-average CAPs. Although this masking procedure was not strictly required and did not affect the general observations in this study, it was found to result in slightly more specific CAP patterns (Figure S2).

The number of clusters *k* was set to 30 after comparing the outcomes of setting *k* equal to 20, 30, and 40. Although these three cases generated largely similar CAPs, clustering with *k* = 30 led to a few distinct CAPs that were not found with *k* = 20, while *k* = 40 led to several CAPs that were nearly indistinguishable. Thus, the choice of *k* = 30 was a compromise between extracting too many and too few distinct CAPs base on what was afforded by the data.

### Occurrence rate of CAPs

For each fMRI session, the number of fMRI time frames belonging to a specific CAP was divided by the total number of frames in order to quantify the occurrence rate of the CAP in a subject (only one session for each participant). A permutation test was applied to determine whether the occurrence rates of the CAPs were significantly different between male and female subjects. Specifically, the 102 subjects were randomly assigned to two groups with size of 38 and 64, respectively, without considering their gender, and the difference in CAP occurrence rate between the two groups was recorded. This process was then repeated 50,000 times to build up distributions of the between-group difference for each CAP. The probabilities (*p*-values) of seeing differences between the male and female groups were then calculated by comparing the actual observations to these distributions, and a Bonferroni correction was applied to correct for multiple comparisons.

Head motion was also quantified for the male and female groups based on two metrics (mean translation, mean rotation) calculated from the 6 affine motion parameters. The mean translation was defined as the average displacement between any two consecutive volumes (defined as the root-mean-square of the translation parameters), and the mean rotation was calculated in similar way using the rotation parameters (Van Dijk et al., 2012).

### Correlation maps

For comparison, seed-based correlation maps were calculated by correlating fMRI signals from all brain voxels to those from four seed regions (6 × 6 × 6 mm^3^ cubes) centered at the following locations (with coordinates given in MNI space): posterior cingulate cortex [PCC, (0, −53, 26)], medical prefrontal cortex [mPFC, (0, 52, -6)], left intraparietal cortex [IPS, (−24, −58, 52)], and motor cortex [(−36, −25, 57)], respectively (Van Dijk et al., 2010).

### Spatial ICs and temporal functional modes (TFMs)

As another methodological comparison, group-level spatial ICA with temporal concatenation was implemented with the MELODIC program in the FSL software (Beckmann et al., 2005). The number of components to be extracted was specified as 30, matching the number of the clusters specified for the clustering. The CAPs were also compared to 21 TFMs derived previously by temporal ICA (Smith et al., 2012), which are available at SumsDB (http://sumsdb.wustl.edu/sums/directory.do?id=8288032&dir_name=TFM_PNAS).

For comparison, the CAPs and ICs/TFMs that have highest spatial correlation to each other among their own category were paired up and shown in Figure 6 and Figure S7.

## Results

### Activity distribution at single time frames

**Figure 1:**
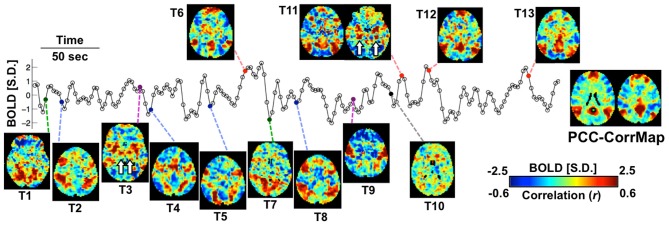
**Spontaneous activity at single fMRI time frames is suggestive of varying network involvement**. Sample frames (T1-T13) from a representative fMRI scan show clear, distinct spatial patterns, some of which (red points) resemble the DMN pattern demonstrated by the PCC-based correlation map of this subject (right). The fMRI time course represents signal from the PCC seed region (green square). Small structures indicated by white arrows are the hippocampus (HI) and posterior parahippocampal gyrus (PHG) in T11 and the ventral lateral nucleus (VL) in T3. The color bar represents normalized BOLD signal for the sample frames and correlation values for the seed-based correlation map.

Exemplary activity time frames (T1-T13) from a resting-state fMRI scan are shown in Figure 1, together with a map derived by temporally correlating signals across the brain with that from a “seed” region in the PCC. While the correlation map highlights the PCC, medial frontal cortex (MF), and bilateral parietal cortices (LPC), all recognized as primary nodes of an RSN known as the “default mode” network (DMN) (Greicius et al., 2003), single time frames often show clearly deviating patterns. For example, while T6, T11, T12, and T13 resemble DMN, T1 and T7 show co-activations at the visual cortex; T3 and T9 have high signal level specifically at the sensorimotor and insular cortex; T2, T4, T5, and T8 cover different sub-sections of the intraparietal sulcus (IPS) as well as some frontal areas, including the frontal eye fields (FEF); at T10, no apparent co-activation pattern is seen. It is worth noting that some of the patterns include strong co-activation at small but very specific brain structures, e.g., the hippocampus (HI) and posterior parahippocampal gyrus (PHG) in T11 and the ventral lateral nucleus (VL) of the thalamus in T3 (white arrows in Figure 1). Although some of these differences may be manifestations of experimental noise, their spatial characteristics (e.g., bilaterality and anatomical specificity) are suggestive of a neuronal origin.

### Spontaneous co-activation patterns (CAPs)

To characterize stable, recurring co-activation patterns in spontaneous activity, single fMRI time-frames (13382 frames total) were clustered into 30 groups based on their spatial characteristics, after which within-group averaging was performed to calculate canonical CAPs (see Methods). These 30 CAPs were ranked by their similarity values, defined as the average correlation between each fMRI time frame within a group and their mean (Figure S3). Many of these CAPs resembled RSNs extracted with conventional analysis, suggesting network activity in the DMN, sensory regions with or without DMN involvement (see examples in Figure S3).

**Figure 2:**
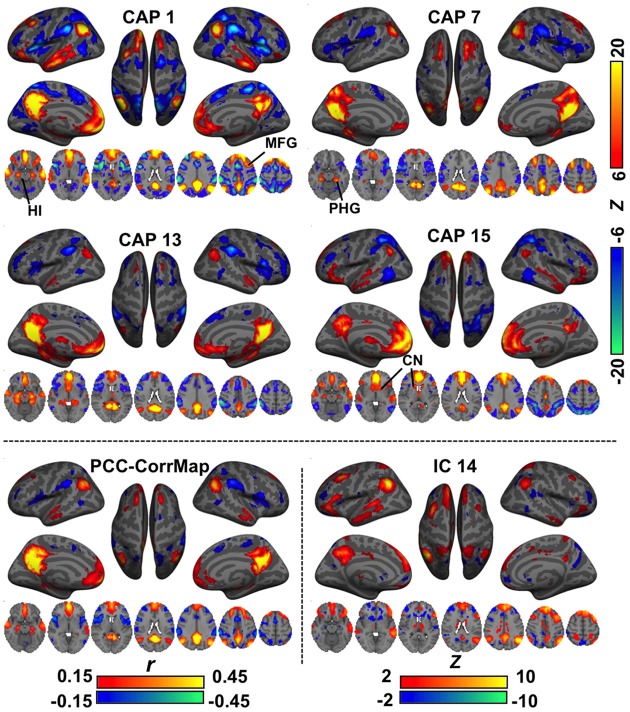
**Comparison of CAPs with the DMN derived from conventional analysis methods**. CAPs 1,7,13,15 coarsely resemble the DMN pattern, but differ in detail. For example, CAP 1, 3, and 15 show specific co-activations at the hippocampus (HI), parahippocampal gyrus (PHG), and caudate nucleus (CN), respectively. For comparison, the DMN was also derived from PCC-based correlation (PCC-CorrMap, bottom left) and ICA (IC 14, having the highest spatial correlation with the PCC-CorrMap, bottom right). Both methods failed to extract multiple patterns of DMN seen in the CAPs.

In various brain regions, CAPs showed interesting differences from RSNs (Figure 2). For example, DMN CAPs 1 and 15 selectively involved the HI, CAP 7 selectively involved the posterior PHG, and CAP 13 involved both. This distinct involvement of HI and PHG in DMN is not observed with conventional analysis. In addition, CAP 1 showed an asymmetric activation at the superior portion of the left middle frontal gyrus (MFG) even though its general pattern is fairly symmetric between hemispheres; CAP 7 has very specific activation along the superior frontal gyrus (SFG), and its LPC components localize more posteriorly compared to the others; and CAP 15 shows clear co-activations at the caudate nucleus (CN). Moreover, co-activations at the PCC/Precuneus regions in CAPs 7 and 13 appear to be patterned differently from those in CAPs 1 and 15. All these DMN-related CAPs are also associated with strong de-activations in a set of “task-positive” regions (Fox et al., 2005), although to varying spatial extent.

**Figure 3:**
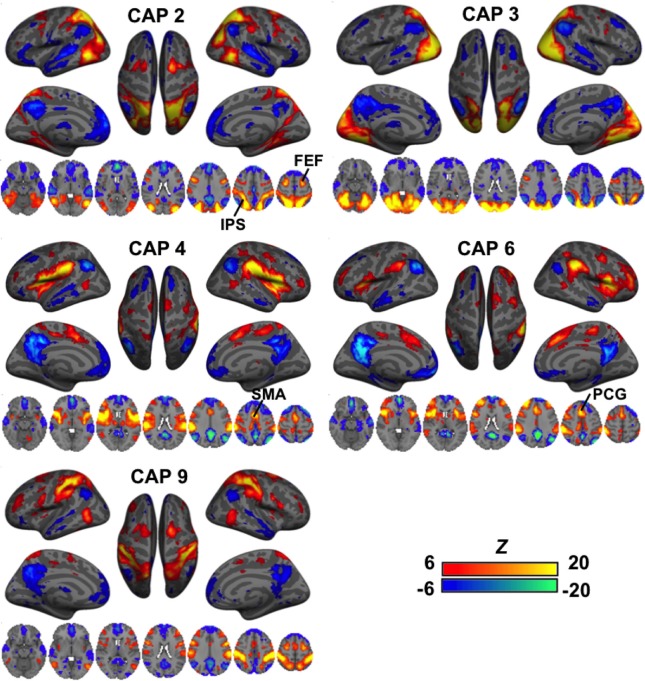
**CAPs showing distinct co-activation in “task-positive” regions (i.e., activated with common tasks) but consistent de-activation in the “task-negative” areas (i.e., de-activated with common tasks)**. Their patterns appeared more distinct from one another as compared to the DMN-activated CAPs. While CAPs 2 and 3 are both related to the visual system, CAP 2 overlaps more on the high order visual area and frontal eye fields (FEF). CAPs 4 and 6 looks almost identical in some axial slices, but a close inspection reveals that CAP 4 covers the central opercular cortex (CO), parietal operculum (PO), insular, thalamus, and supplementary motor area (SMA), whereas CAP 6 involves more anterior regions, including the frontal operculum (FO) and paracingulate gyrus (PCG). Moreover, even though all these CAPs show activations in the IPS, they cover very different sub-sections.

A second group of CAPs showed de-activations in DMN-related regions combined with strong co-activations in a set of “task-positive” regions (Figure 3). Their patterns appeared more distinct from one another as compared to the DMN-activated CAPs. While CAPs 2 and 3 are both related to the visual system, CAP 2 overlaps more on the high order visual area and FEF. CAPs 4 and 6 looks almost identical in some axial slices, but a close comparison reveals that CAP 4 covers the central opercular cortex (CO), parietal operculum (PO), insular, thalamus, and supplementary motor area (SMA), whereas CAP 6 involves more anterior regions, including the frontal operculum (FO) and paracingulate gyrus (PCG). Moreover, even though all these CAPs show activations in the IPS, they cover very different sub-sections. There are also CAPs showing highly lateralized patterns (Figure S4) or strong activation in the primary sensory regions (Figure S5).

**Figure 4:**
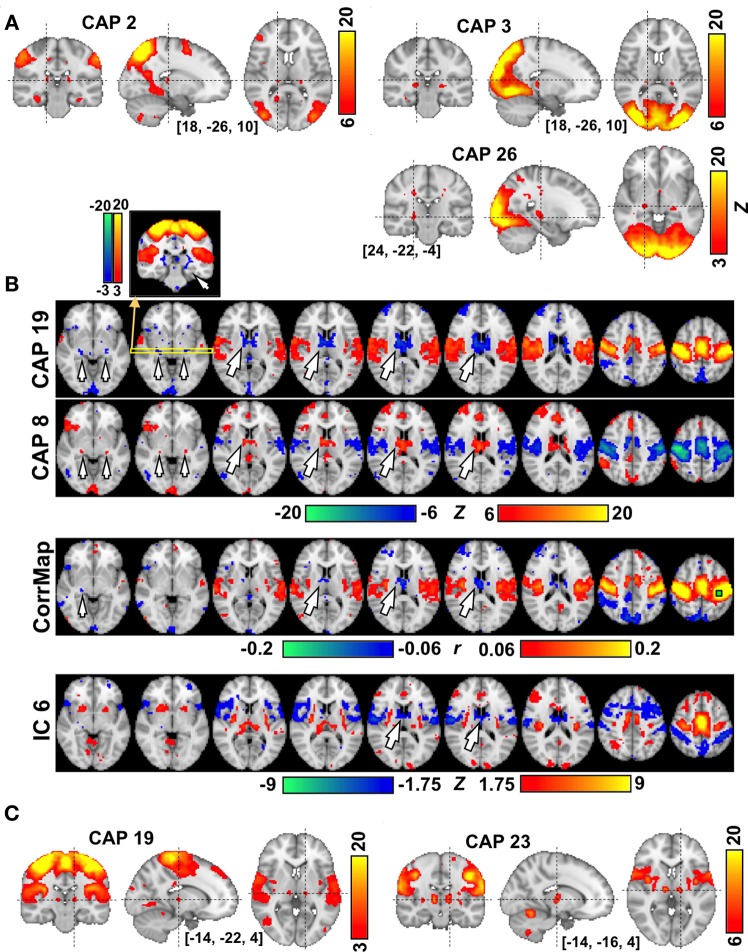
**The involvement of thalamic nuclei in CAPs. (A)** CAPs in visual areas show co-activations in the LGN (CAP 26) and in different subdivisions of the Pulvinar (CAPs 2-3) each with different involvement of visual cortex. **(B)** CAPs in sensori-motor areas showing anti-phase activity at the AN/MDN of the thalamus (big arrows) and some areas surrounding the posterior thalamus (small arrows). The correlation map (CorrMap) seeded in the motor cortex (green square) and IC 6 less clearly shows anti-phase activity in these thalamic regions. Note: the display thresholds for CorrMap and IC 6 were adjusted in order to display approximately the same number of negative voxels as in CAP 19. **(C)** Sensorimotor-related CAPs also show co-activations at the thalamic nuclei, including the VPL (CAP 19) and VL (CAP 23), which are separated by only a few millimeters.

Several CAPs showed focal activity in thalamic and cerebellar structures. For example, CAPs 2 and 3, both of which involve higher order visual and visual association areas, include spatially distinct thalamic nuclei, with the ones in CAP 2 located more superior, medial, and anterior than those in CAP 3 (Figure 4A). In both CAPs, this thalamic activity appears to occur within the pulvinar. In contrast, CAP 26, whose activity shows a preference for primary visual cortex, includes bilateral focal structures that are much more inferior, lateral, and anterior than the thalamic structures seen in CAPs 2 and 3. We tentatively attribute these focal structures to the lateral geniculate nuclei (LGN). In contrast to the vision-related CAPs, thalamic nuclei in sensorimotor-related CAPs tend to show activity opposite to the cortical regions. Strong de-activations (or activations) are observed in the anterior and medial dorsal nuclei (AN/MDN, big arrows in Figure 4B), and in some regions surrounding posterior thalamus (small arrows), when the sensorimotor cortex shows activation (resp. de-activation) in CAP 19 (resp. CAP 8). These regions are much less distinct (for the AN/MDN) or even absent (for the thalamus surrounding areas) in maps derived by seed-based correlation or ICA (note: the display thresholds for these latter maps were adjusted, base on the whole brain, to include approximately the same spatial extent of significantly negative regions). It should also be noted that a pair of thalamic nuclei showing co-activation with cortical regions could also be clearly seen in CAP 19 when lowering the display threshold (Figure 4C). This pair of nuclei is attributed to the ventral posterolateral nuclei (VPL), located about 6 mm posterior to the ventral posteromedial nucleus (VL) shown in CAP 23 (Figure 4C).

The 30 CAPs are available at our website (http://amri.ninds.nih.gov/pub/xiao/CAPs_30_2mm.nii.gz).

### Occurrence rate of CAPs

An interesting aspect of the proposed analysis approach is that it not only provides spatial maps of co-activating brain regions, but also allows extraction of incidence rates of spatially distinct co-activations, information that is not explicitly available with conventional analysis methods. This incidence or occurrence rate of CAPs may facilitate distinction between subject populations. To illustrate this, we examined the possibility of distinguishing between spontaneous brain activity in males (*n* = 38) and females (*n* = 64) based on differences in occurrence rates of all 30 CAPs. For this purpose, the occurrence rate of a CAP was determined from the fraction of total volumes that were classified into the CAP's associated cluster.

**Figure 5:**
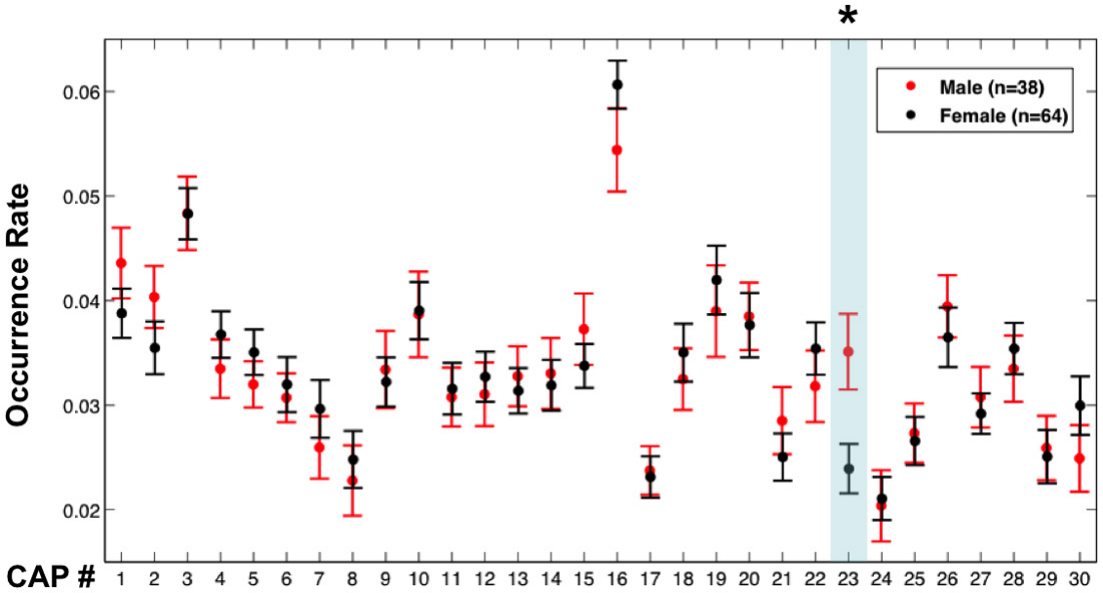
**Occurrence rates of the CAPs in males and females**. Error bars represent the standard error across participants. The asterisk and light blue shadow indicates CAPs showing a significant difference in their occurrence (*p* < 0.01 with Bonferroni correction, permutation test) between these two groups.

CAP 23, which primarily covered sensorimotor areas corresponding to head regions, was found to occur more frequently (*p* < 0.01, Bonferroni corrected, permutation test) in the male members than in the female members (Figure 5). However, the males (mean motion: 0.048 ± 0.021 mm) had a little more (*p* = 0.038, 2-sample *t*-test) head motion than the females (mean motion: 0.040 ± 0.018 mm), which may have affected this result (Van Dijk et al., 2012). To exclude this confounding factor, the same comparison was repeated between 30 males and 39 females whose mean motions exceeded 0.03 mm, which resulted in subgroups with insignificant difference in their mean motion (*p* = 0.25) (we were unable to generate subgroups with similar member counts based on upper threshold for motion). These two subgroups continued to show a significant difference in occurrence rates of CAP 23 (Figure S6).

### Comparison to other data-driven approaches

**Figure 6:**
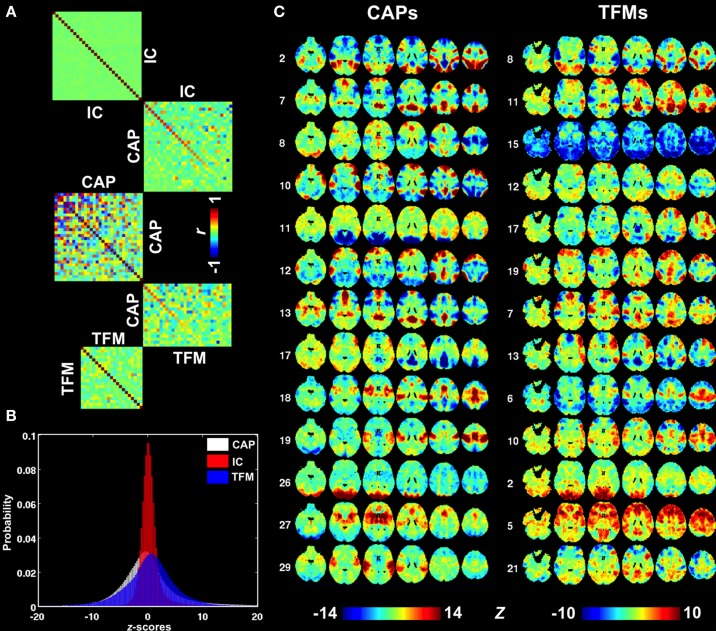
**Comparison between CAPs, ICs, and TFMs. (A)** Spatial cross correlation matrices indicating substantial differences between CAPs (middle) on one hand and ICs and TFMs on the other (top and bottom, respectively). Cross-modality correlations were sorted based on correlation strength. **(B)** Distributions of map statistics (z-statistics) for CAPs (white), ICs (red), and TFMs (blue). IC statistics show a very non-Gaussian distribution with a elevated right tail, consistent with the independence assumption of ICA. **(C)** A selection of best-matched CAPs and TFMs, based on spatial similarity.

A more comprehensive comparison of CAPs with independent components (ICs) derived from the same dataset is presented in Figure S7 and Figures 6A,B. ICs typically cover a small but specific set of brain areas with very high z-statistics. As a result of the spatial independence criterion, there is little overlap of the regions having high z statistics in different ICs (Figure S7), resulting in a spatial correlation very close to zero (Figure 6A). In contrast, several CAPs, particularly those related to DMN or task-positive regions (Figure S3, CAPs 1,7,12,15, and CAPs 2,3,4,6,9, respectively), cover relatively large brain areas and show substantial spatial correlations with one another (Figure 6A). The CAPs in this category do not have clear correspondence to the patterns seen in the 30 ICs. In contrast, the CAPs covering small but specific regions, e.g., those related to primary sensory systems, tended to more closely resemble ICs (Figure S7, CAPs 16,19,23,26,29). Judging from their mutual spatial cross-correlation, at least a third of the CAPs did not pair up with any of the ICs (Figure 6A). Another noticeable difference between CAPs and ICs is asymmetric tails observed for the distribution of ICA map statistics (Figure 6B), which corresponds to the lack of significantly negative values in the ICs (Figure S7).

Recently, temporal ICA was applied to the components generated from spatial ICA in order to generate a set of temporal functional modes (TFMs) (Smith et al., 2012). Similarity between TFMs and CAPs may be expected, since temporal independence between TFMs is, to some degree, similar to the exclusive relation of a time point to a specific CAP. To investigate this, we performed a spatial similarity analysis comparing the 30 CAPs with 21 TFMs derived in Smith et al. (2012). Although there were several CAP—TFM pairs with high similarity (CAP 2 and TFM 8, CAP 12 and TFM 19, and CAP 26 and TFM 2), most CAPs did not have a matching TFM (Figures 6A,C).

## Discussion

In this report, we demonstrated a method to extract brain functional information by identifying regions that spontaneously co-activate during resting conditions. The method is based on the notion that co-activation of functionally related brain regions may not be a continual, stationary phenomenon (is implicitly assumed in conventional analysis) but rather occur sporadically over the course of a few seconds. The method also extends its previous version of seed-based analysis targeting specific networks (Liu and Duyn, 2013) to a data-driven, whole-brain approach.

By applying this approach to a large dataset of resting state fMRI studies, we identified at least 30 reproducible, spatially distinct co-activation patterns (CAPs). These CAPs in some aspects resemble canonical RSNs and TFMs extracted with ICA, but also show distinct differences in various brain regions. These differences are partly attributed to methodological differences, for example the fact that the proposed method does not assume statistical independence between CAPs, as is the case for RSNs and TFMs extracted with ICA. Thus, the proposed method may lead to an alternative interpretation of the NNs underlying resting-state fMRI signals.

### Distinct co-activation patterns and dynamic functional connectivity

A series of recent studies has attempted to reveal dynamic changes in RSNs by examining the temporal variability in fMRI signal correlations (Chang and Glover, 2010; Allen et al., 2012; Hutchison et al., 2012; Rack-Gomer and Liu, 2012). Considerable variations in RSN characteristics were found even over the duration of a typical (several minute) resting scan, suggesting that the underlying NNs may dynamically assemble and disassemble over this time window. Such temporal variations may pose significant challenges for correlational approaches for the extraction of RSNs, as it may render them strongly dependent on the choice of analysis window. For example, the PCC-seeded correlation map within a 16.1-s window (only 7 time points) including T11 shows a DMN pattern with high correlations at the HI and the posterior PHG (Figure S8) because they co-activated at T11 (Figure 1); their functional connectivity to the PCC seed, however, nearly vanishes when the time window is shifted forward by 23 s to cover T12 instead. Likewise, the thalamic nucleus seen in T3 is only present in correlation maps seeded at the motor cortex for time windows including T3 (Figure S8). This dependence on position of the analysis window can be mitigated or avoided by increasing the window duration, which leads to more stable, reproducible RSN, but on the other hand can result in the loss of network information contained in single time frames.

### Information in CAPs

Using the method proposed in this study, the analysis window was effectively reduced to include only a single time point, and sensitivity was subsequently improved through selectively averaging the fMRI volumes with similar patterns. By averaging selectively rather than continually (as implicitly occurs with correlation analysis), one may extract finer detail regarding spontaneous co-activations of multiple brain regions. For example, correlation analysis with a seed in the PCC and a typical analysis window of a few minutes generally reveals a functional connection with HI and posterior PHG but little other information. In contrast, the multiple DMN-related CAPs extracted with the proposed method suggests that the two regions at times independently connect to the PCC. A possible explanation for these regions' differential involvement with DMN is their distinct roles in memory storage and retrieval (Gluck and Myers, 1997). Similarly, the varying involvement of sensory regions with the DMN apparent from CAPs (Figure 3) may signify that the brain may not be simply organized into two anti-correlated NNs spanning “task-positive” and “task-negative” regions, as concluded previously from correlation analysis (Fox et al., 2005).

Many CAPs included small structures in the thalamus and cerebellum that appeared to localize to specific anatomic subregions. The vision-related CAPs included thalamic nuclei that were well-separated and corresponded to established targets of functional connections with specific regions of the visual cortex that co-occurred in the CAPs. Similarly, the sensorimotor CAPs corresponding to the head (CAP 23) and lower body (CAP 19) regions showed specific co-activations at the VL and VPL, respectively, despite their limited anatomical separation of only a few millimeters. Anti-phase interaction was also observed between thalamic nuclei and sensorimotor cortex in CAPs 8 and 19. The AN/MDN in these CAPs has been shown to positively correlate with the alpha-band (8-13 Hz) electroencephalography (EEG) power, which is, in turn, negatively correlated with multiple sensory regions, including the sensorimotor cortex (Liu et al., 2012). These thalamic regions have non-specific connections to the cortex and may participate in the process of alertness and arousal (Van Der Werf et al., 2002). The second set of thalamic regions showing negative correlation to the sensorimotor cortex was around the LGN. Lowering the display threshold exposed multiple diffuse areas encompassing the posterior thalamus, which we suspects to be the thalamic reticular nucleus (TRN), partly because the anti-phase activity with sensorimotor cortex is, to some extent, consistent with the established inhibitory influence of TRN over other thalamic nuclei.

A few CAPs included isolated cortical structures that were distant from regions of major activity. For example, CAP 16, which includes areas associated with peripheral vision, showed small co-activation in the SMA, motor cortex, and medial IPS. This is plausible considering the critical role of the medial IPS in visuomotor coordinate transformation (Grefkes et al., 2004). These subtle aspects of CAPs are generally not captured with ICA or correlation analysis, indicating potential advantages of the proposed analysis approach.

Since hemodynamic response function (HRF) has been demonstrated to vary spatially (Wu et al., 2013), a potential concern is that different CAPs may actually represent the same neuronal co-activation event at different hemodynamic delays. This is, however, unlikely for the following two reasons. First, the fact that CAPs, particularly those covering similar or close cortical regions, show specific co-activations in distinct thalamic and/or cerebellar structures and that many of these CAP patterns are consistent with known organization of the brain argue strongly against their origin being attributable to vascular effects (e.g., hemodynamic delay differences between regions). Secondly, the analysis of temporal precedence of CAPs failed to find any pair of CAPs occurring in a specific order (Figure S9), which is also inconsistent with the confounding effect of the HRF delays. Conversely, co-activation in the same CAP does not guarantee the exact synchronization of neural activity at corresponding regions. Therefore, the “co-activation” in CAP refers to synchronization of fMRI signals rather than that of the underlying neural activity.

We found both co-activation and co-deactivation CAPs for some brain regions (e.g., CAP 8 and CAP 19), which are actually averages around fMRI signal peaks and troughs, respectively. Although these two types of CAPs often show very similar spatial patterns with reversed sign, a close inspection revealed that the co-activation at the fMRI peaks is significantly more synchronized than the co-deactivation at the troughs (Figure S10). The implication of this observation remains unclear, but the peaks and troughs are clearly corresponding to distinct cortical states.

An attractive aspect of the proposed analysis is that it provides a simple quantifiable measure of CAP occurrence rate that facilitates comparison between subject populations. As example of this, we demonstrated that CAP 23, which overlaps the sensorimotor cortex, occurs more frequently in males than in females. Although far from providing a physiological explanation, this finding is consistent with a previous study reporting sex differences in the fluctuation amplitude of resting-state fMRI signals in similar brain regions (Biswal et al., 2010). We also make publicly available the 30 CAPs derived in our study, which can be directly used as templates for future studies intending to examine and compare their occurrence rates in different groups or under different conditions.

### Comparison with conventional methods

As mentioned above, the extraction of CAPs based on single-time frame analysis may allow one to better capture temporally localized spontaneous co-activations as compared to conventional correlation analysis. The method presented here resembles spatial ICA in the sense that both are spatial-domain methods that regard fMRI time frames as the basic units of analysis. However, ICA assumes that the fMRI volumes comprise weighted combinations of a set of statistically ICs, the best fit of which can be found by maximizing the independence of the latter. The independence assumption yields ICs that typically cover a relatively small extent of the brain with very high statistical scores (Figure S7), because such a pattern has high non-Gaussianity (Figure 6B), an important factor underlying ICA based signal separation. At the same time, independence maximization yields ICs with minimal pair-wise spatial correlations, as shown in Figure 6A. Together, these two features suggest that the ICs present a spatial parcellation of the brain rather than distinct states of functional connectivity. In addition, ICs may exhibit considerable pairwise *temporal* correlation as exemplified by the often negative correlation observed between the ICs covering task-positive and task-negative regions.

To overcome these limitations and gain more knowledge about the brain's spontaneous co-activations, temporal ICA has been further applied to spatial ICs to recombine them into a set of TFMs (Smith et al., 2012). The TFMs are perhaps more closely related to the CAPs, since the concept of temporal independence is similar to the exclusive presence of a given CAP at any given time. However, a direct comparison between TFMs and CAPs indicated substantial differences (Figure 6). This may not be surprising, given the rather different methodology and assumptions used to generate these network measures.

A practical advantage of the proposed method is that it involves few assumptions and transformations of the data. The classification procedure (clustering) does not perform any transformation on the data, and the CAPs are simply averages of time frames classified into groups based on spatial activity patterns. Therefore, they are easy to interpret and reflect the underlying brain activity in a rather direct way.

### Neural origin of CAPs

Since the co-activation patterns are clear even in single fMRI volumes, they likely reflect large-scale neural activity occurring within brief time periods. One candidate is large-scale neuronal avalanching activity, defined as spontaneous activity initiating at a specific location in the form of a brief burst, and taking with it, like an avalanche, other connected regions that are near activation threshold (Beggs and Plenz, 2003; Tagliazucchi et al., 2012). The previous analysis on spatiotemporal dynamics of spontaneous fMRI signals confirmed its scale-free property, consistent with avalanche types of activity at smaller time-scales (Tagliazucchi et al., 2012). However, it is also possible that other types of neuronal activity underlie such brief co-activations of the brain. For example, since the dataset we analyzed here was acquired during wakefulness, it is possible that neural activity associated certain conscious processes, e.g., mind wandering, may also contribute to spontaneous co-activations of the brain. The analysis of fMRI signals acquired under brain states with reduced consciousness would help to partially clarify these issues. Nevertheless, uncovering the precise neuronal origin and functional relevance of the CAPs will likely require acquisition of electrophysiological data concurrently acquired with the fMRI.

### Technical limitations

A challenge for clustering analysis is selection of *k*, i.e., the number of CAPs to be extracted from the data. The current value of 30 was chosen somewhat arbitrarily from a comparison of results obtained with *k* = 20, 30, and 40. We have also attempted to select *k* by evaluating the corresponding cluster structures with more objective indices, including the Silhouette, Calinski-Harabasz, Davies-Bouldin methods, which, however, yielded inconsistent recommendations that also tend to underestimate the number of CAPs present in the data. This may be due to the two following reasons: first, the distance (dissimilarity) among the CAPs is likely to have a very skewed distribution, with a portion of the CAPs being much closer to one another than to the others (Figure 6A), which increases the difficulty in finding a clear division. Secondly, the noisy fMRI volumes mentioned above will further blur the boundaries between different CAPs. Nevertheless, the CAPs in the current dataset were found to be rather insensitive to this parameter, as the majority of the CAPs shown for the case of *k* = 30 were also observed in the cases of *k* = 20 and *k* = 40.

The computational load of the *k*-means clustering increases quickly with the number of fMRI volumes (time points). Therefore, the application of the proposed method to fMRI datasets with very high sampling rate, e.g., those acquired with the newly developed multi-band technique, would either be limited to a relatively small population or have to require more computational resources and/or more efficient computational algorithms. This could be a challenge for future studies attempting to utilize the ultra-high temporal resolution of fMRI data for special research purposes.

### Conflict of interest statement

The authors declare that the research was conducted in the absence of any commercial or financial relationships that could be construed as a potential conflict of interest.
