# Supplementary material for: Decomposition of spontaneous brain activity into distinct fMRI co-activation patterns
Source: Front Syst Neurosci. 2013 Dec 4;7:101. doi: 10.3389/fnsys.2013.00101 (PMC3913885; doi:10.3389/fnsys.2013.00101)
Supplement: Supplementary file 1 [file Presentation1.PDF]

Decomposition of Spontaneous Brain Activity into Distinct fMRI Co-activation Patterns

Xiao Liu*, Catie Chang, and Jeff H. Duyn

Supplementary Figures 1−10

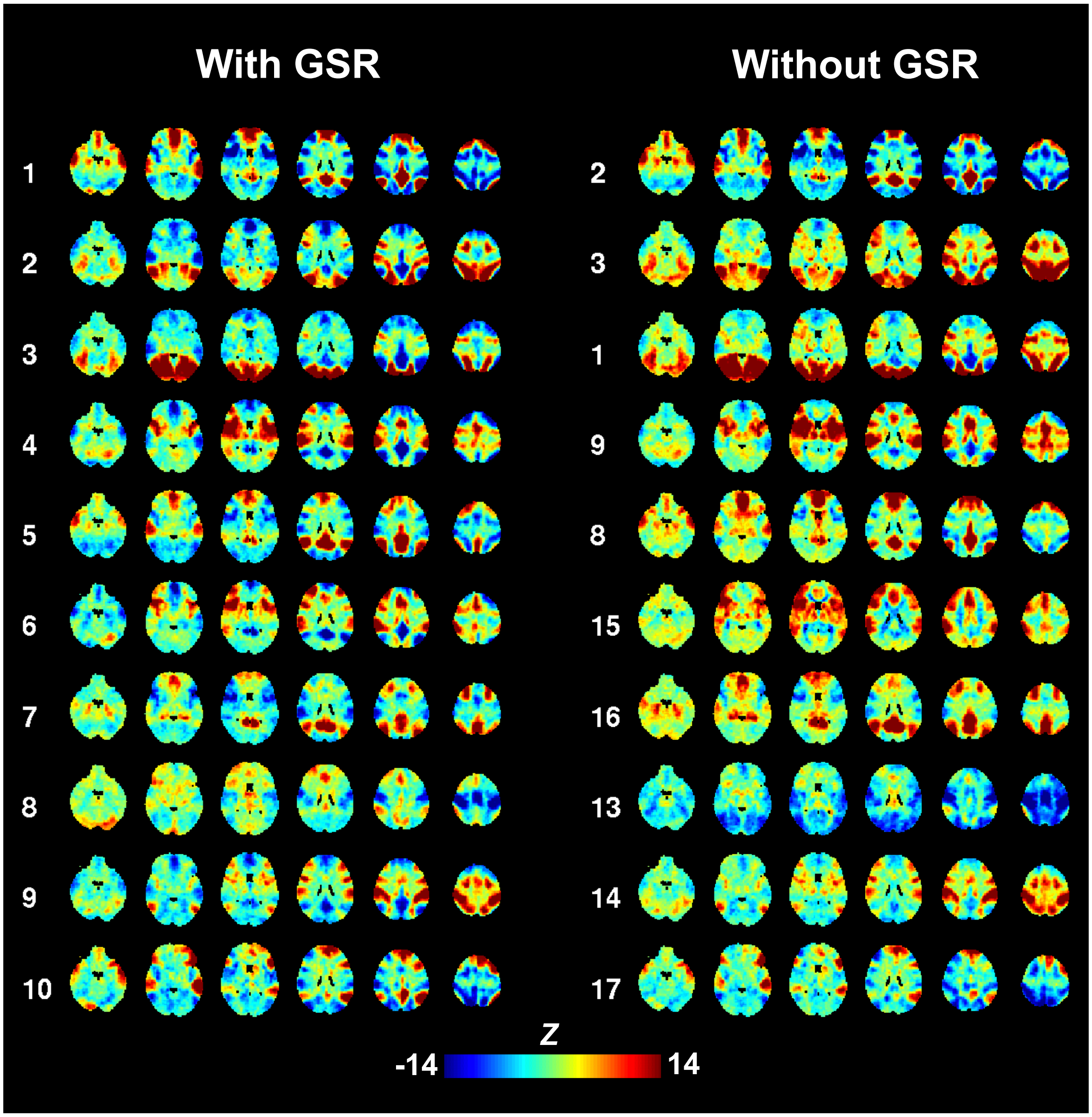

Fig. S1. Effects of global signal regression (GSR) on CAPs. The CAPs 1−10 identified with using GSR are largely similar to corresponding CAPs extracted without using the GSR, except that some CAPs show a global shift of the distribution of their map statistics toward more positive or negative values.

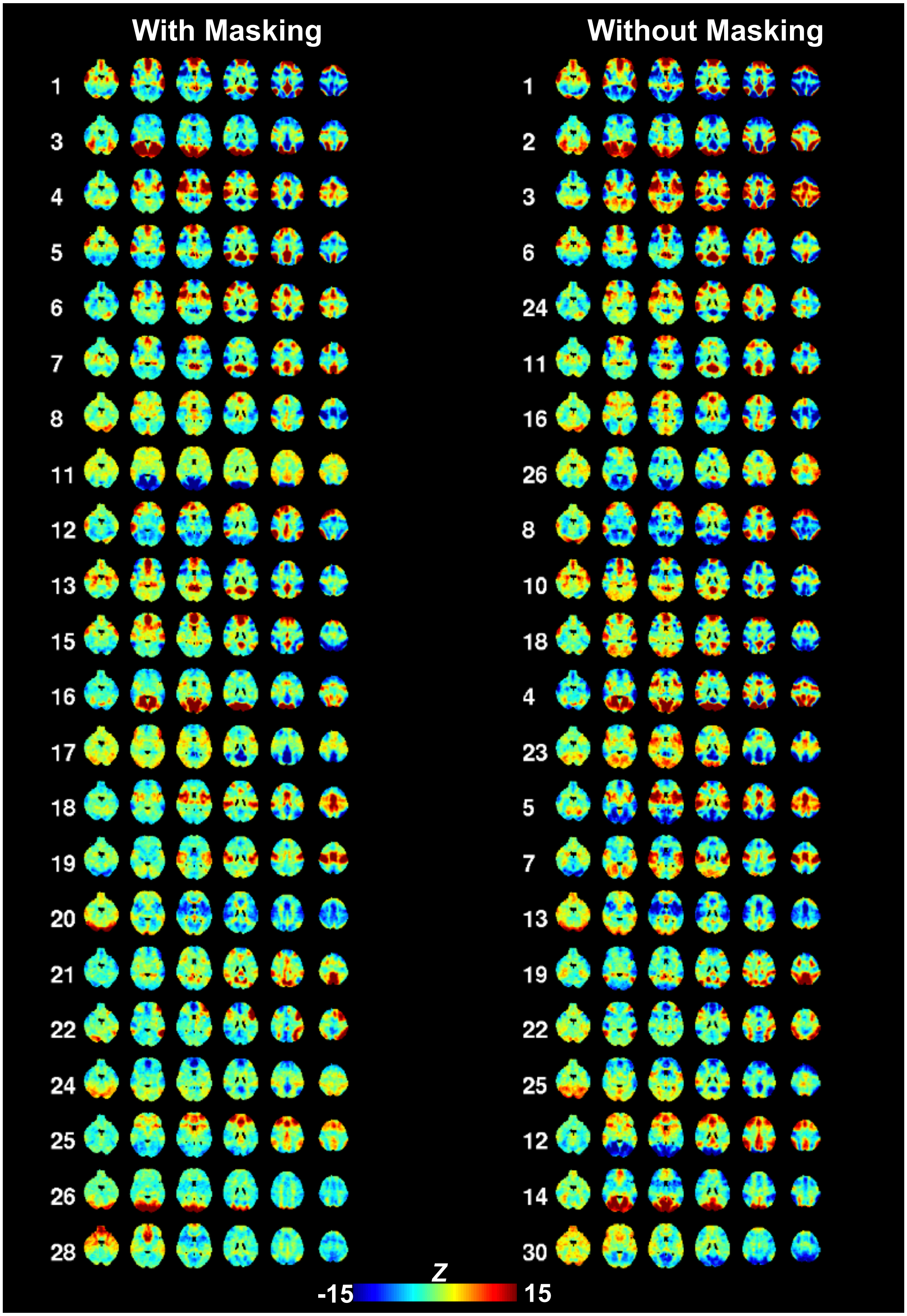

Fig. S2. CAPs obtained with (left) and without (right) noise masking procedure. Only subtle difference are observed between these condition, suggesting noise masking is not critical.

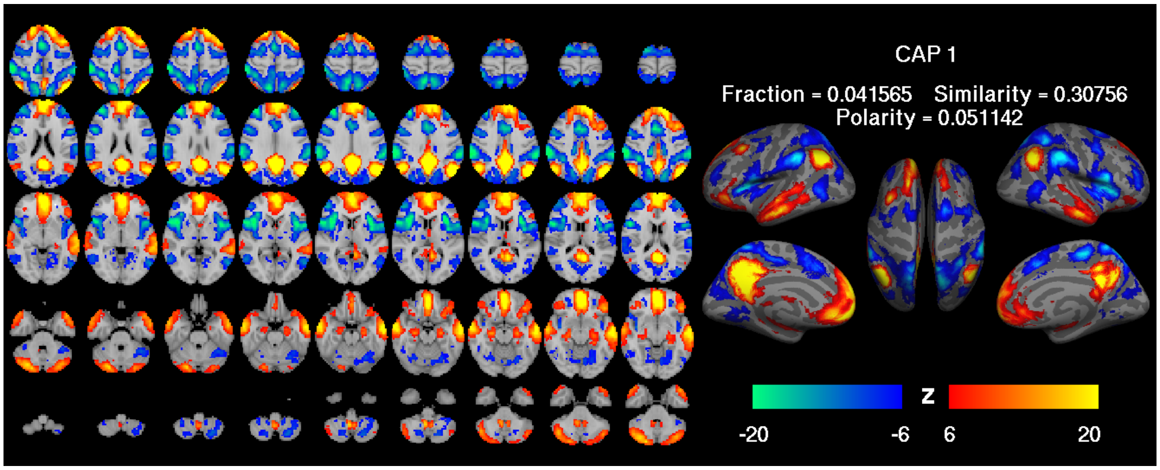
CAP 1
Fraction = 0.041565 Similarity = 0.30756
Polarity = 0.051142
Z
-20
-6
6
20

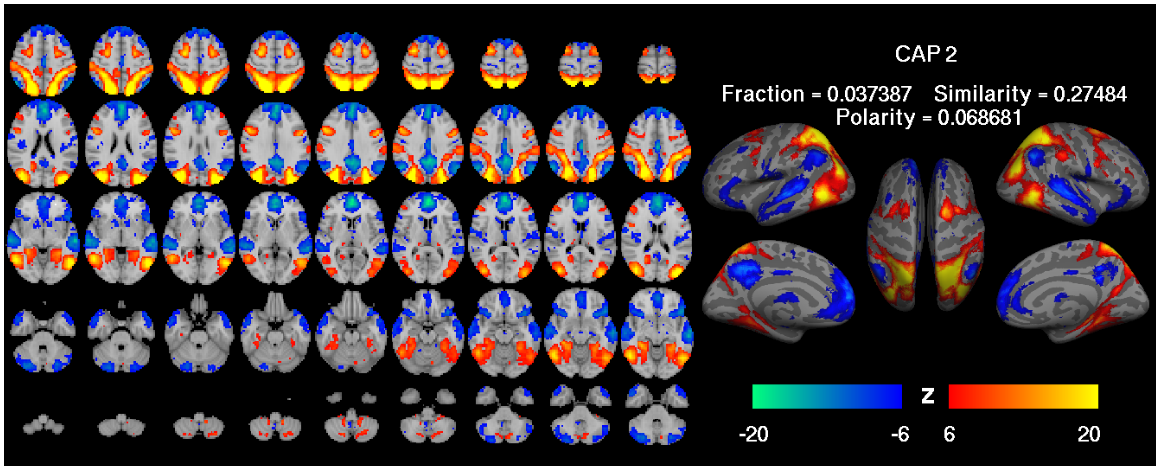
CAP 2
Fraction = 0.037387 Similarity = 0.27484
Polarity = 0.068681
Z
-20
-6
6
20

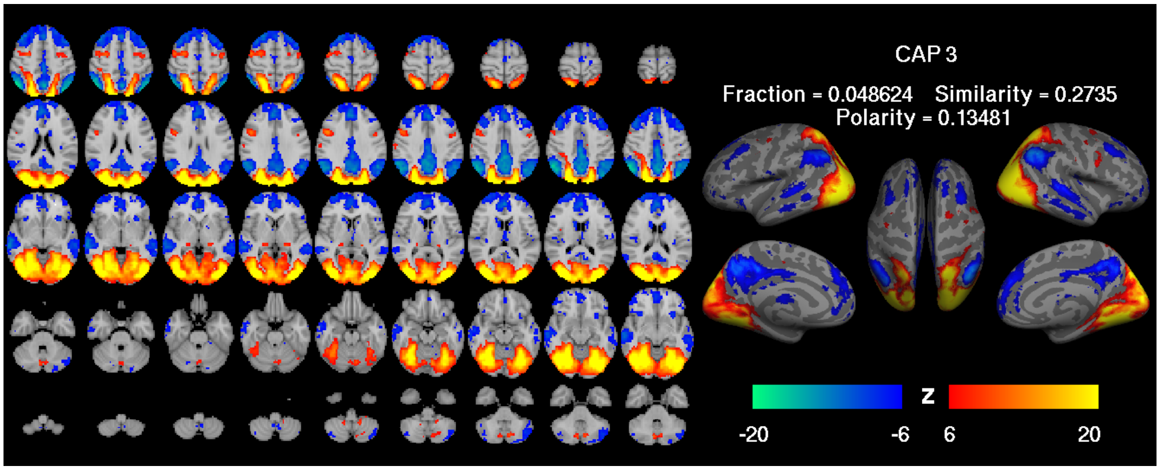
CAP 3
Fraction = 0.048624 Similarity = 0.2735
Polarity = 0.13481
Z
-20
-6
6
20

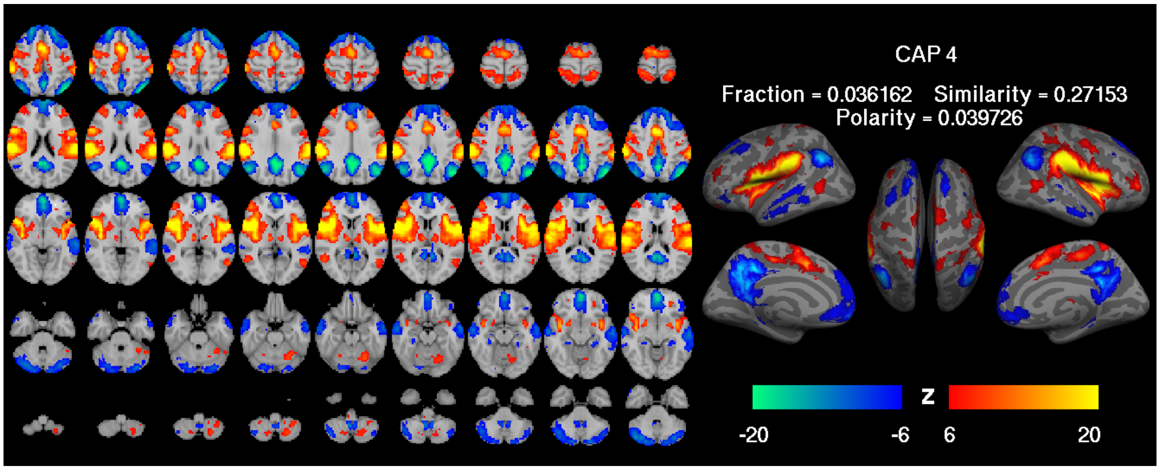
CAP 4
Fraction = 0.036162 Similarity = 0.27153
Polarity = 0.039726
Z
-20
-6
6
20

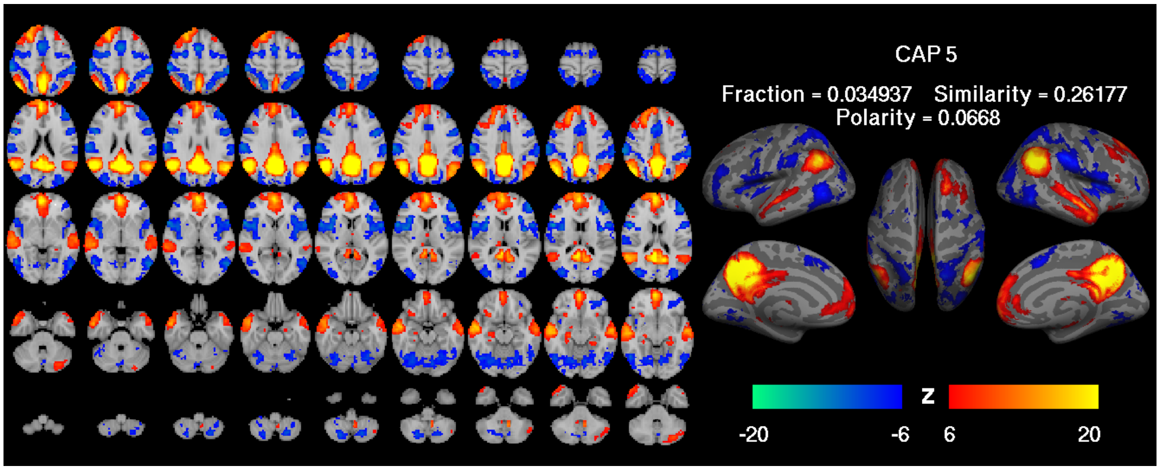
CAP 5
Fraction = 0.034937 Similarity = 0.26177
Polarity = 0.0668
Z
-20
-6
6
20

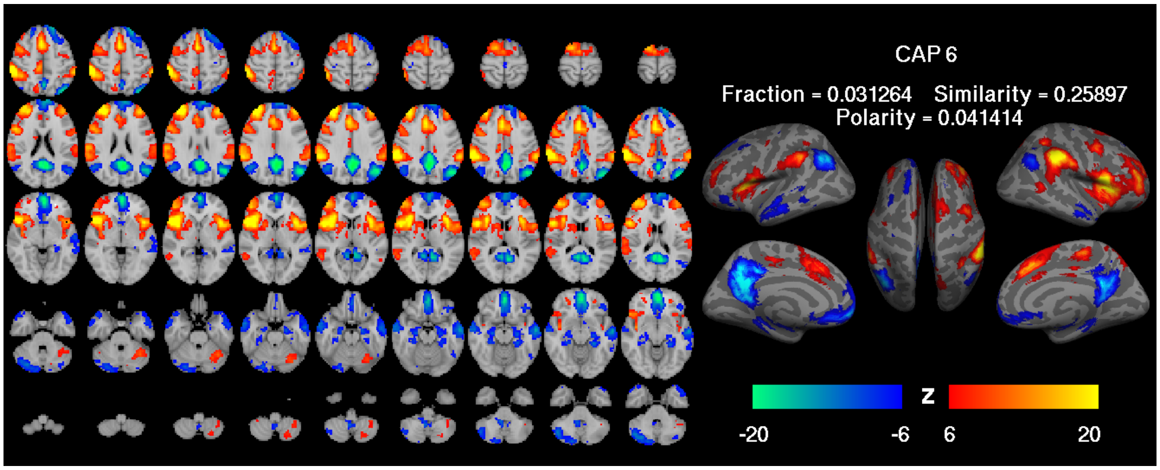
CAP 6
Fraction = 0.031264 Similarity = 0.25897
Polarity = 0.041414
Z
-20
-6
6
20

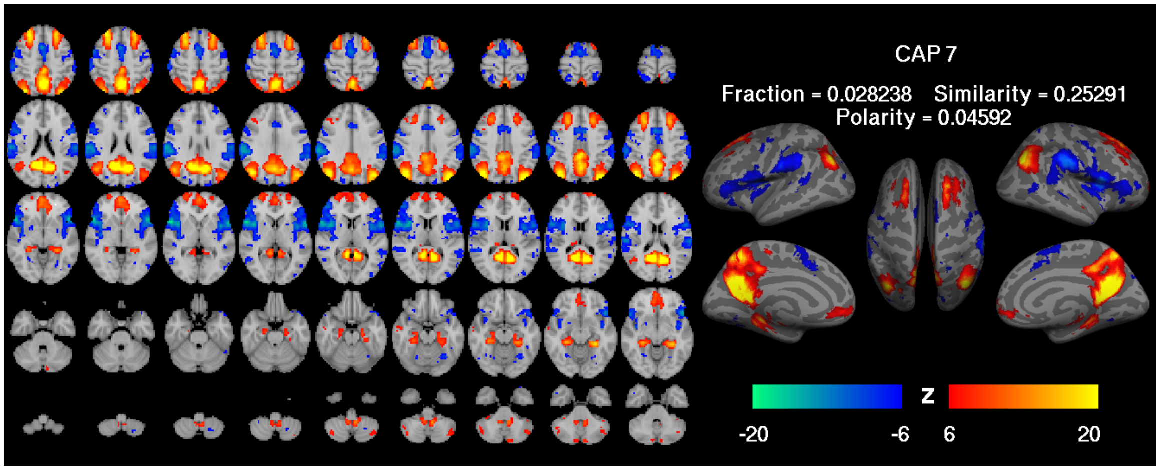
CAP 7
Fraction = 0.028238 Similarity = 0.25291
Polarity = 0.04592
Z
-20
-6
6
20

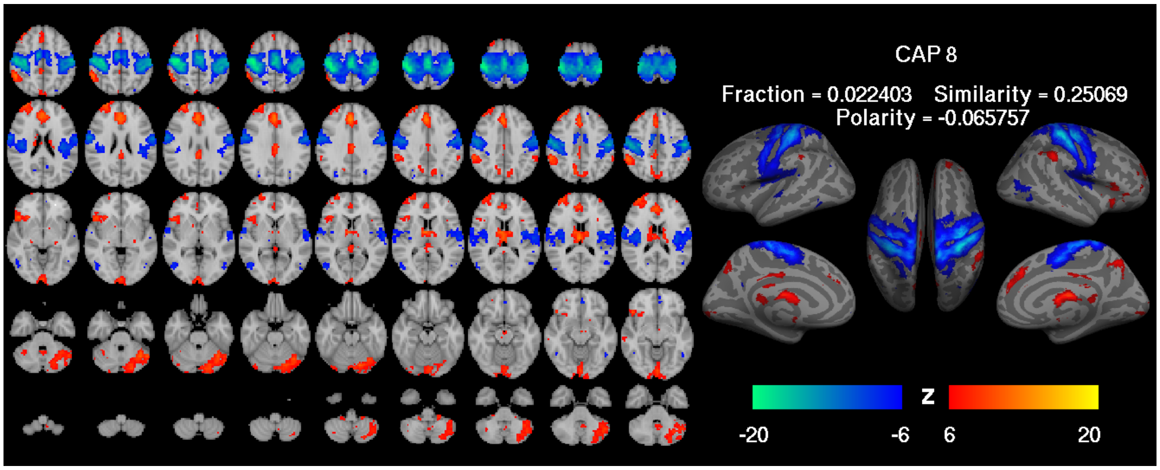
CAP 8
Fraction = 0.022403 Similarity = 0.25069
Polarity = -0.065757
Z
-20
-6
6
20

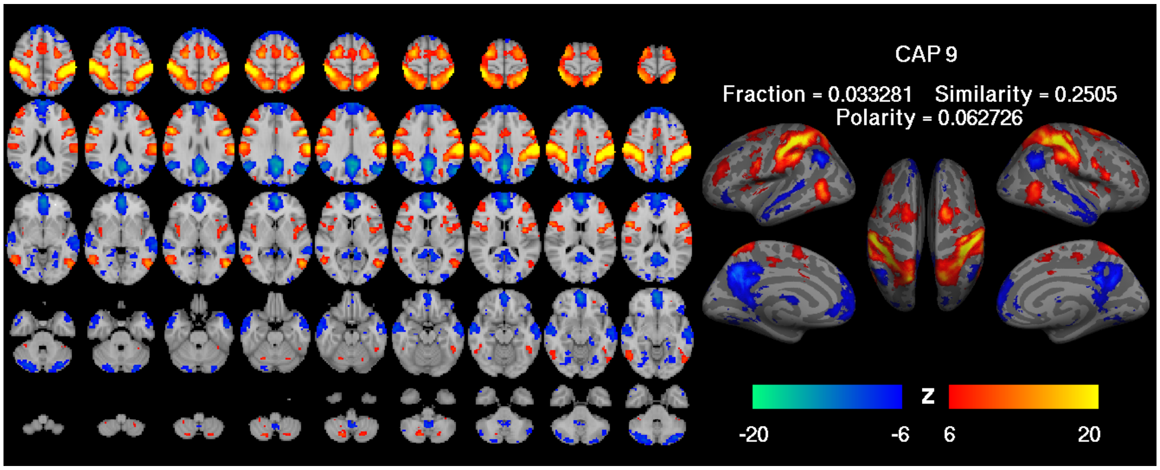
CAP 9
Fraction = 0.033281 Similarity = 0.2505
Polarity = 0.062726
z
-20
-6
6
20

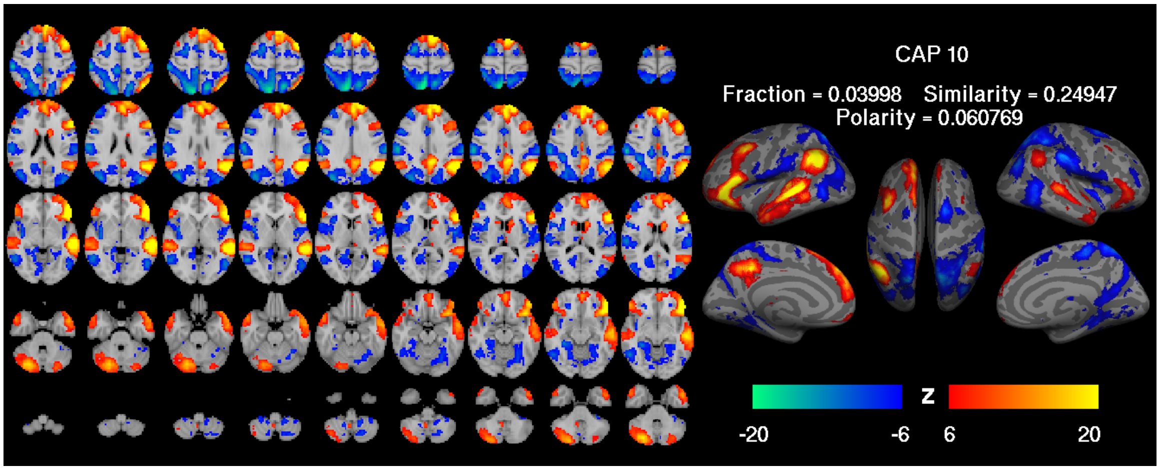
CAP 10
Fraction = 0.03998 Similarity = 0.24947
Polarity = 0.060769
z
-20
-6
6
20

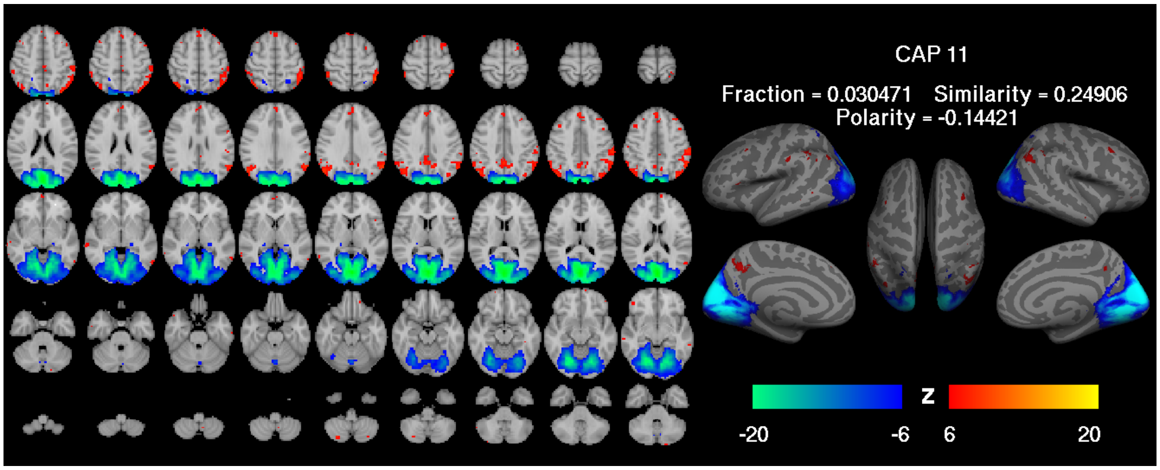
CAP 11
Fraction = 0.030471 Similarity = 0.24906
Polarity = -0.14421
z
-20
-6
6
20

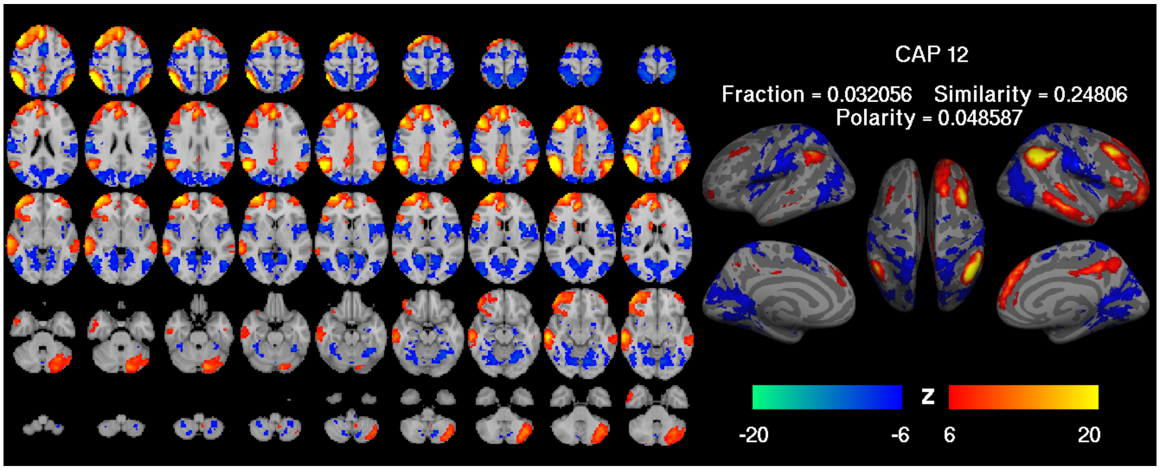
CAP 12
Fraction = 0.032056 Similarity = 0.24806
Polarity = 0.048587
z
-20
-6
6
20

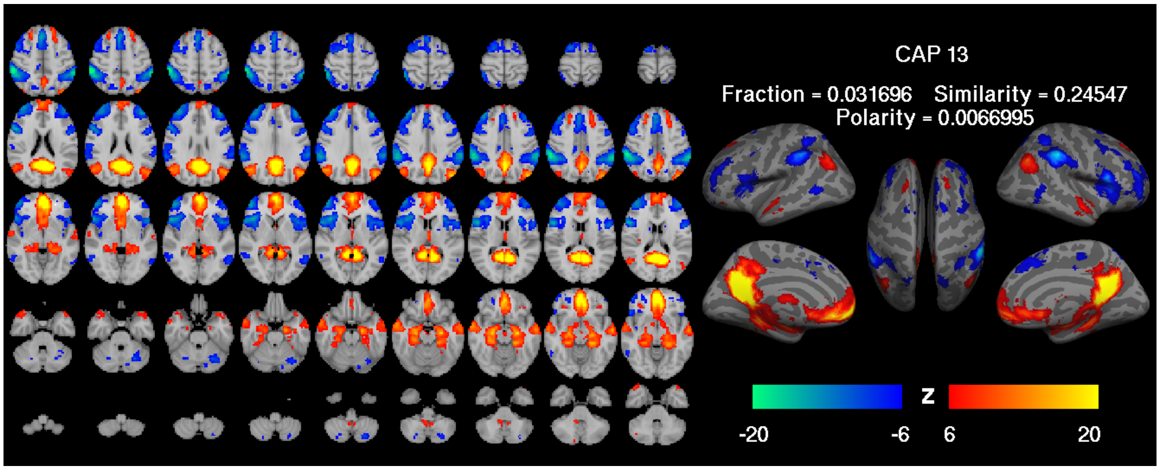
CAP 13
Fraction = 0.031696 Similarity = 0.24547
Polarity = 0.0066995
z
-20
-6
6
20

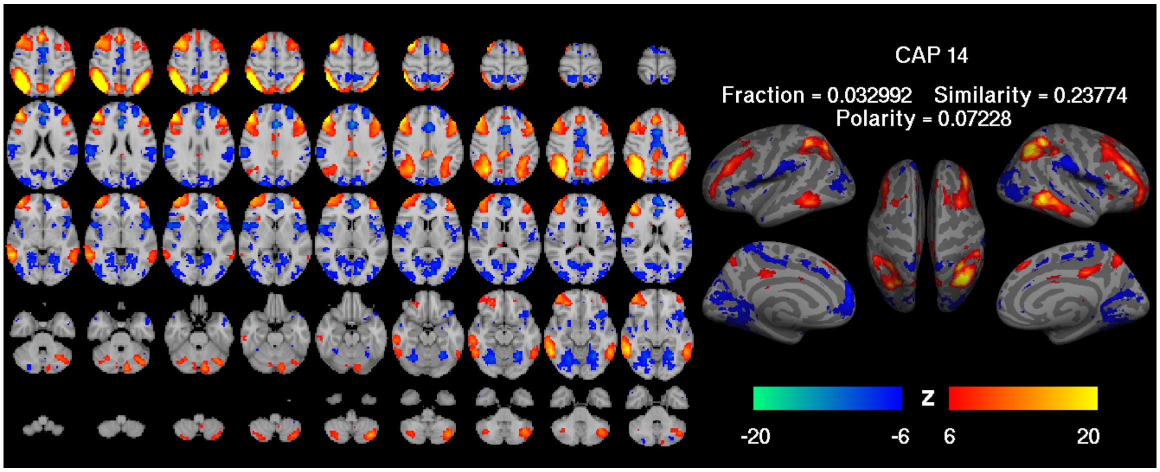
CAP 14
Fraction = 0.032992 Similarity = 0.23774
Polarity = 0.07228
z
-20
-6
6
20

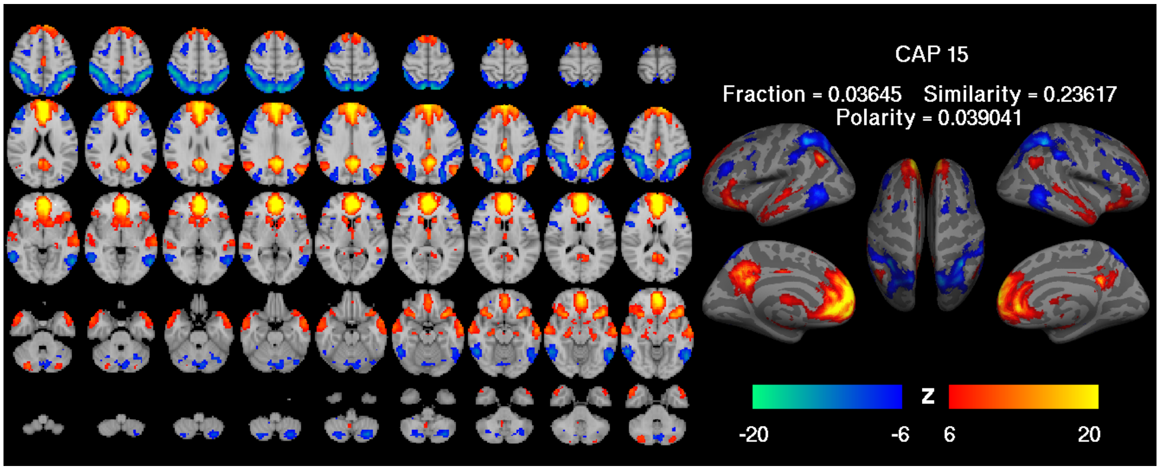
CAP 15
Fraction = 0.03645 Similarity = 0.23617
Polarity = 0.039041
z
-20
-6
6
20

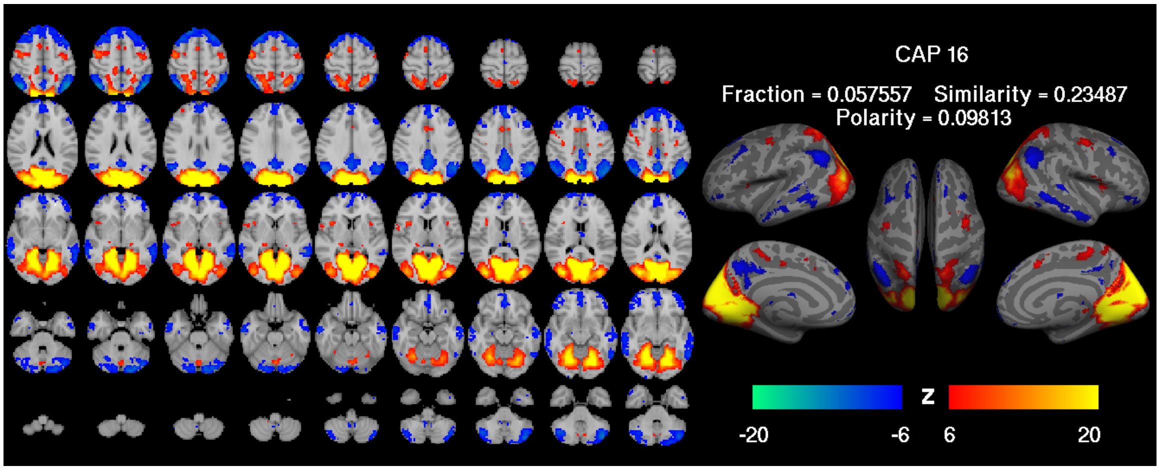
CAP 16
Fraction = 0.057557 Similarity = 0.23487
Polarity = 0.09813
z
-20
-6
6
20

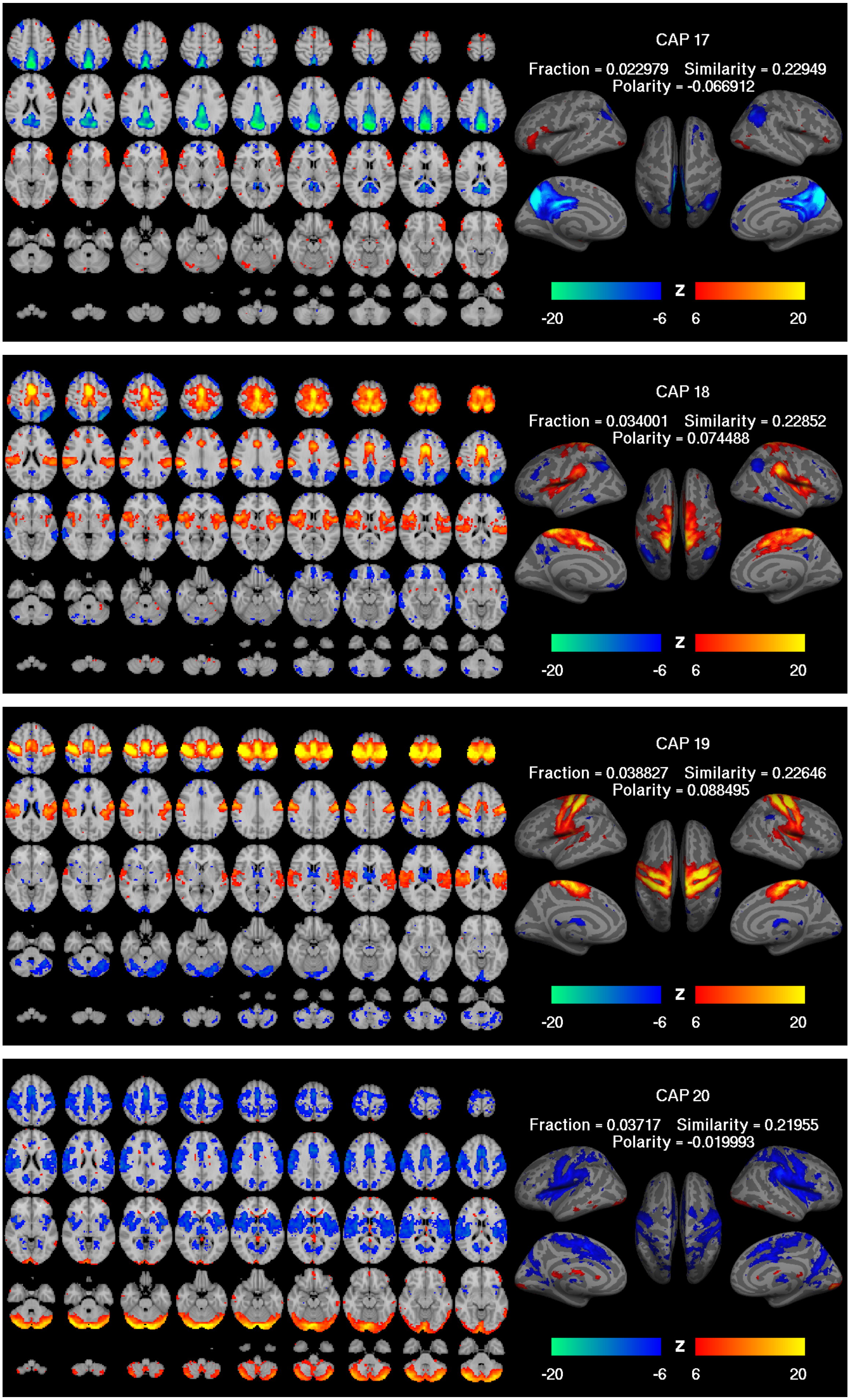
CAP 17
Fraction = 0.022979 Similarity = 0.22949
Polarity = -0.066912
Z
-20
-6
6
20
CAP 18
Fraction = 0.034001 Similarity = 0.22852
Polarity = 0.074488
Z
-20
-6
6
20
CAP 19
Fraction = 0.038827 Similarity = 0.22646
Polarity = 0.088495
Z
-20
-6
6
20
CAP 20
Fraction = 0.03717 Similarity = 0.21955
Polarity = -0.019993
Z
-20
-6
6
20

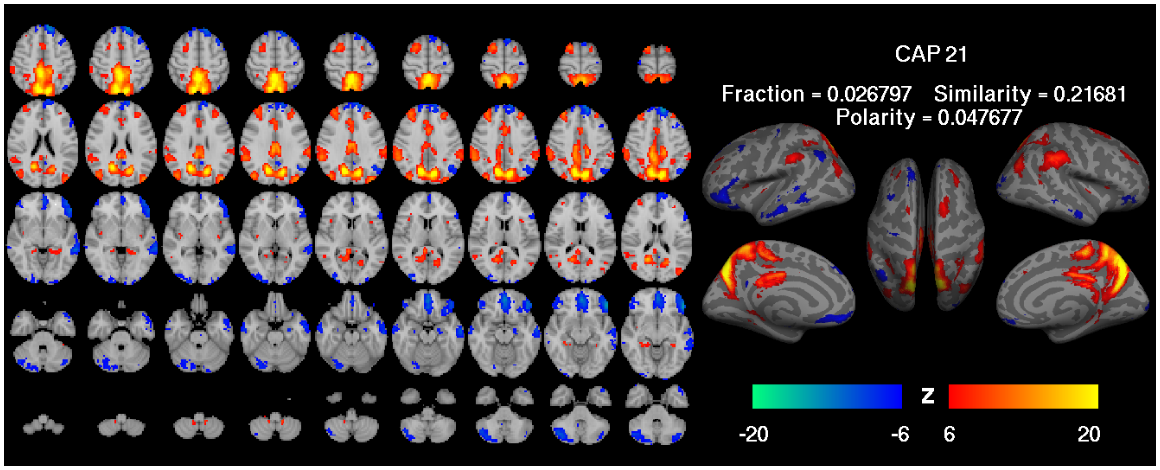
CAP 21
Fraction = 0.026797 Similarity = 0.21681
Polarity = 0.047677
Z
-20
-6
6
20

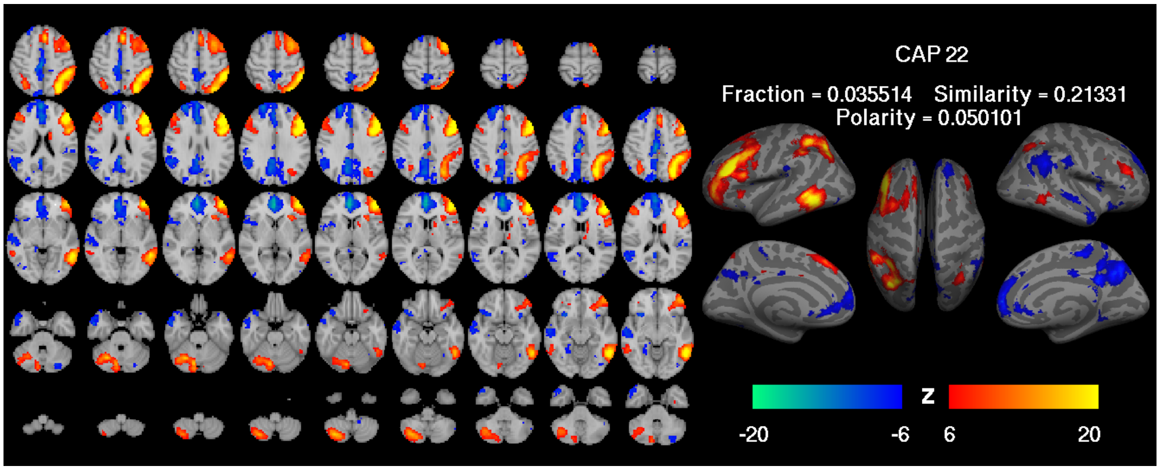
CAP 22
Fraction = 0.035514 Similarity = 0.21331
Polarity = 0.050101
Z
-20
-6
6
20

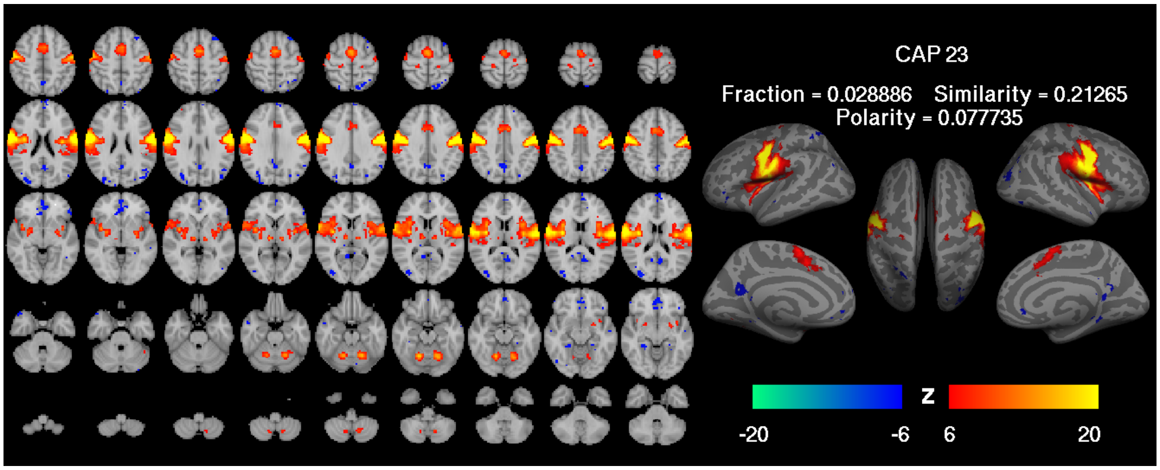
CAP 23
Fraction = 0.028886 Similarity = 0.21265
Polarity = 0.077735
Z
-20
-6
6
20

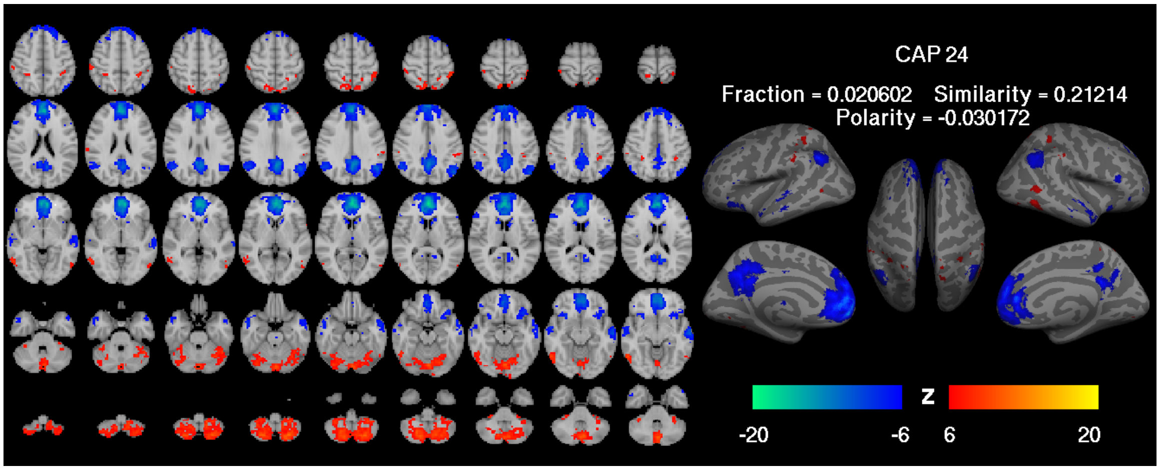
CAP 24
Fraction = 0.020602 Similarity = 0.21214
Polarity = -0.030172
Z
-20
-6
6
20

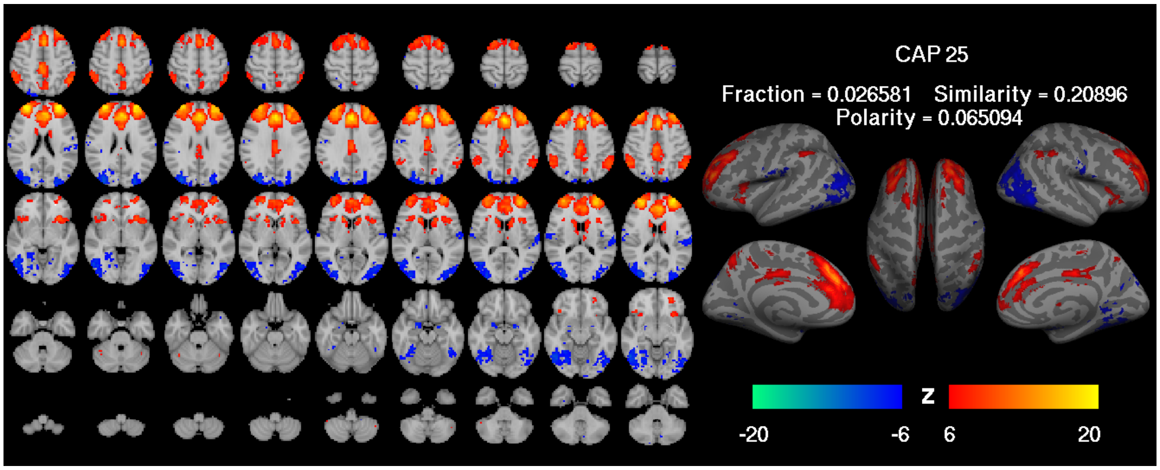
CAP 25
Fraction = 0.026581 Similarity = 0.20896
Polarity = 0.065094
z
-20
-6
6
20

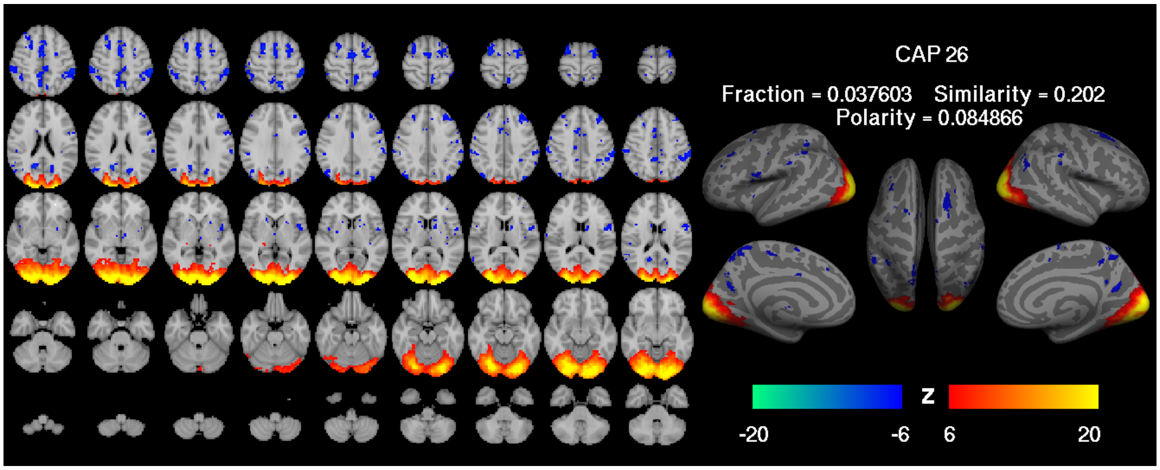
CAP 26
Fraction = 0.037603 Similarity = 0.202
Polarity = 0.084866
z
-20
-6
6
20

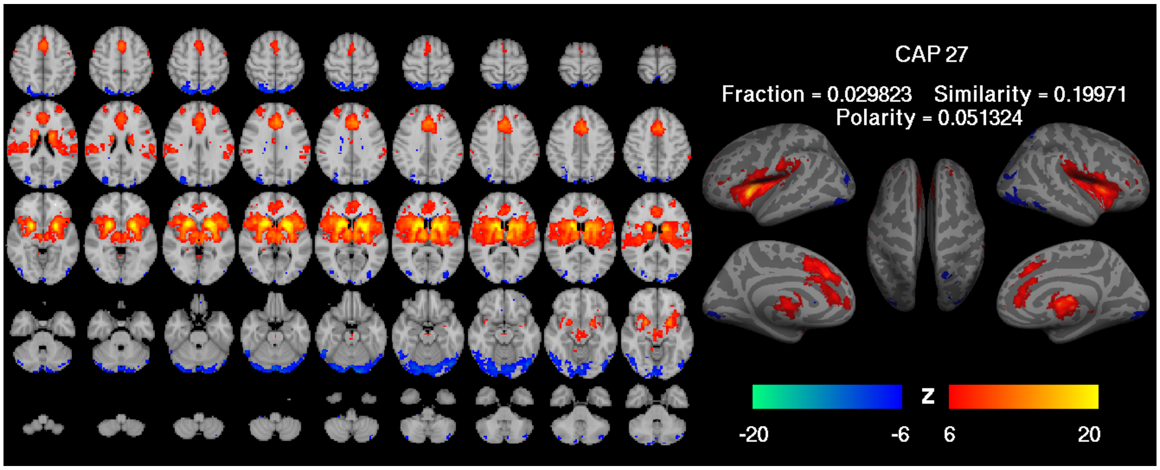
CAP 27
Fraction = 0.029823 Similarity = 0.19971
Polarity = 0.051324
z
-20
-6
6
20

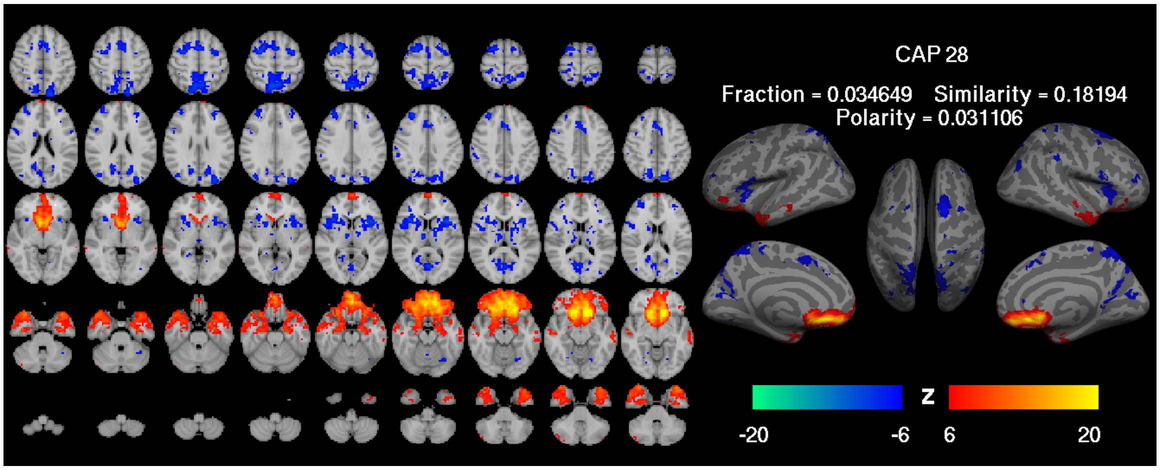
CAP 28
Fraction = 0.034649 Similarity = 0.18194
Polarity = 0.031106
z
-20
-6
6
20

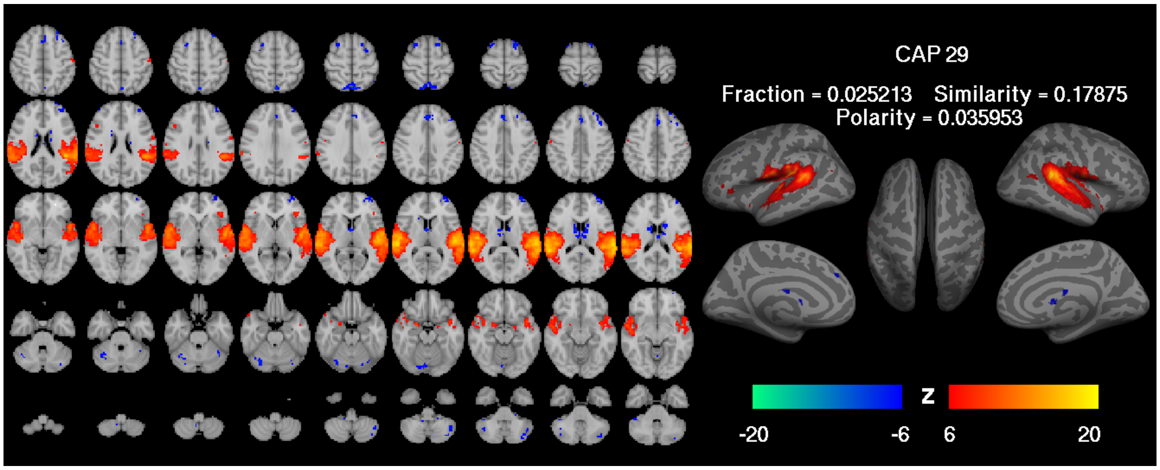

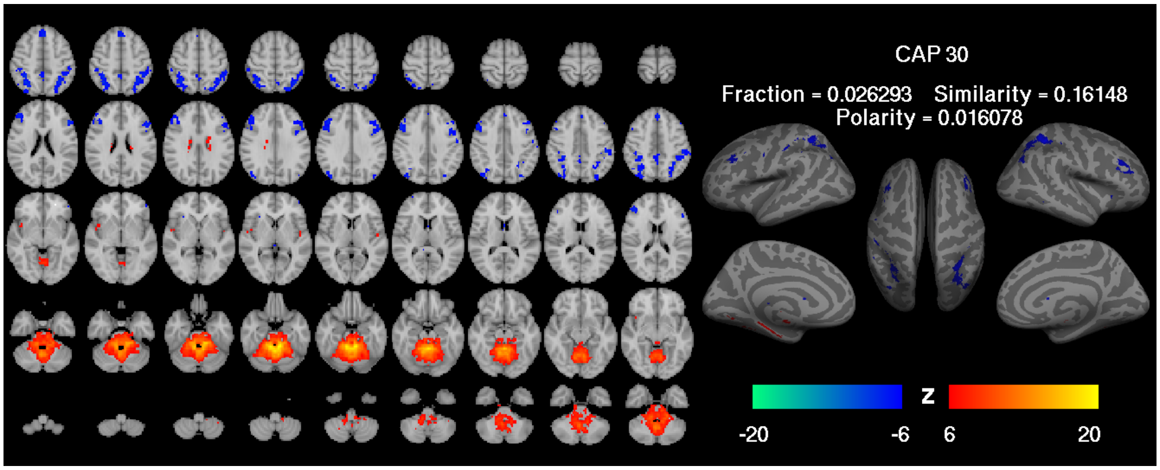

Fig. S3. Detailed information of 30 CAPs extracted with the proposed method. Each CAP was overlaid on the 152-MNI template in 2D slices (upper) and 3D surface (lower) with their occurrence rate, with-group similarity, and polarity showing in the middle. The color encoding of z-score is identical to that in Figure 3 ($6 < |z| < 20$). Many of these CAPs resembled RSNs extracted with conventional analysis, suggesting network activity in the DMN (CAPs 1,7,12,15), sensory regions with or without DMN involvement (CAPs 2,3,4,6,9 and CAPs 16,19,23,26,29 respectively).

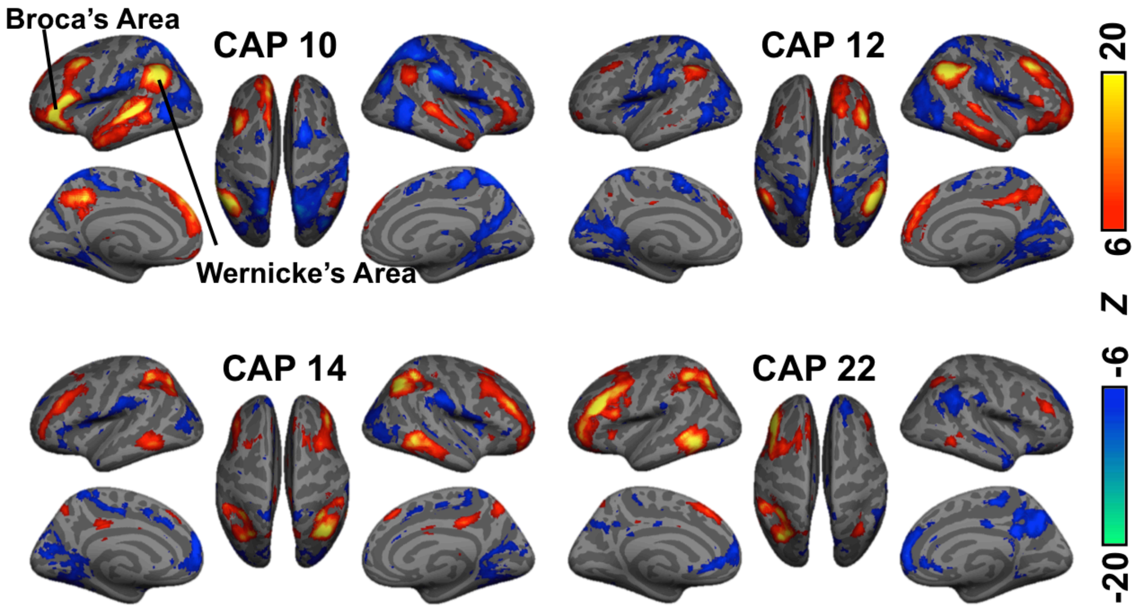

Fig. S4. Several CAPs show highly lateralized patterns. For example, CAP 10 has dominant activation at language-related areas in the left hemisphere, and has a contralateral analogue in CAP 12. Similarly, CAP 22 is lateralized and has its contralateral analogue (although somewhat weakened) in CAP 24. Also worth noting is the fact that the activity patterns in these CAPs often show very sharp transition across the brain's midline, particularly around the PCC and precuneus (Fig. S1, CAPs 10, 12, and 22).

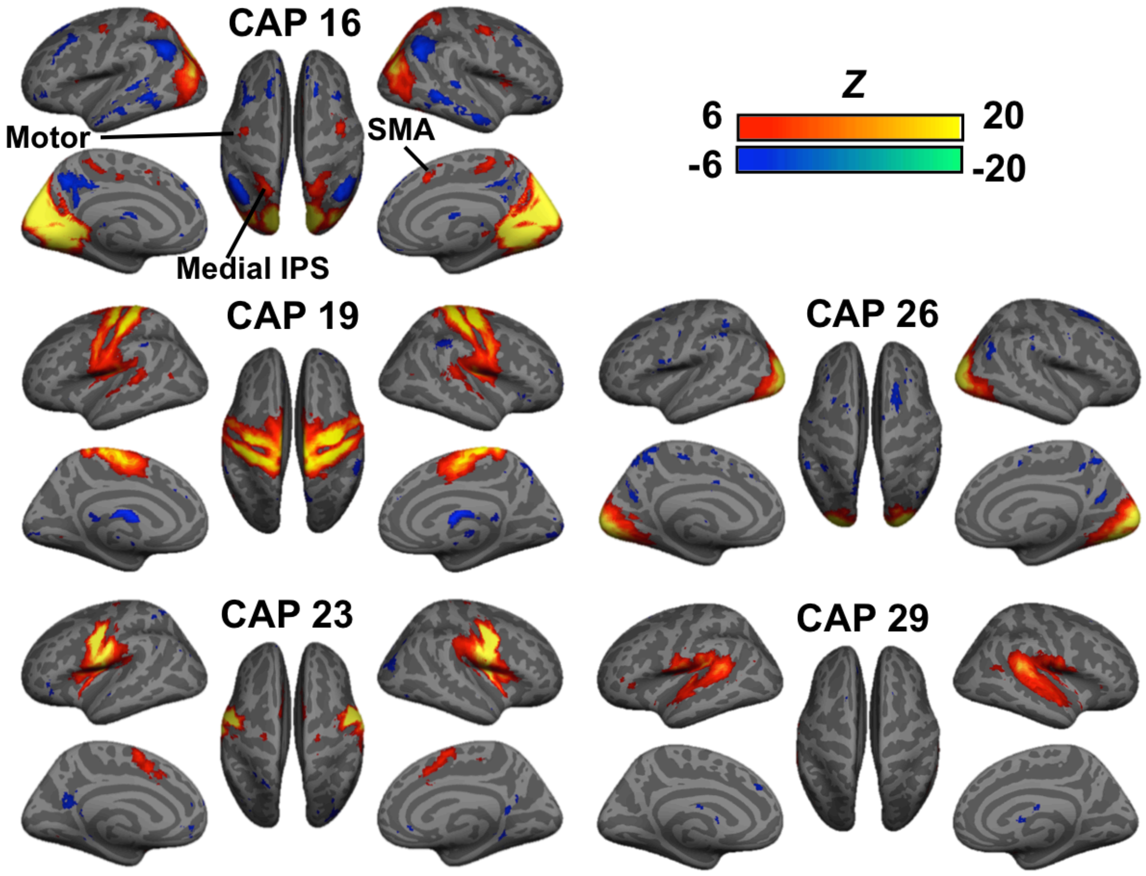

Fig. S5. A number of CAPs exhibited strong activity in primary sensory regions, including the peripheral visual (CAP 16), central visual (CAP 26), auditory (CAP 29), and sensorimotor cortices (CAPs 19 and 23). Of the latter, CAP 19 mainly covers the posterior SMA and the medial sensorimotor regions responsible for lower body parts, while CAP 23 encompasses the anterior SMA and the most lateral sensorimotor regions controlling facial muscles) cortices. While the co-activations in these sensory CAPs are mostly local, some of them show focal structures co-activated in remote brain regions. For example, the visual-related CAP 16 shows very specific co-activations at the motor cortex, SMA, and medial IPS.

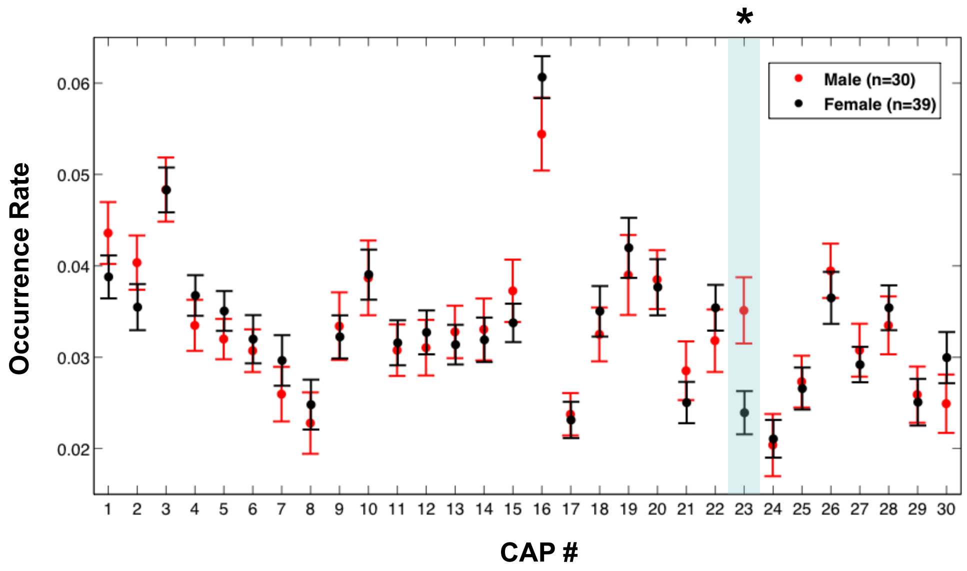

Fig. S6. Occurrence rates of the CAPs in a subgroup of males and females with comparable motions. Error bars represent the standard error across participants. The asterisk indicates CAPs showing a significant difference in their occurrence ($p < 0.01$ with Bonferroni correction, permutation test) between these two groups.

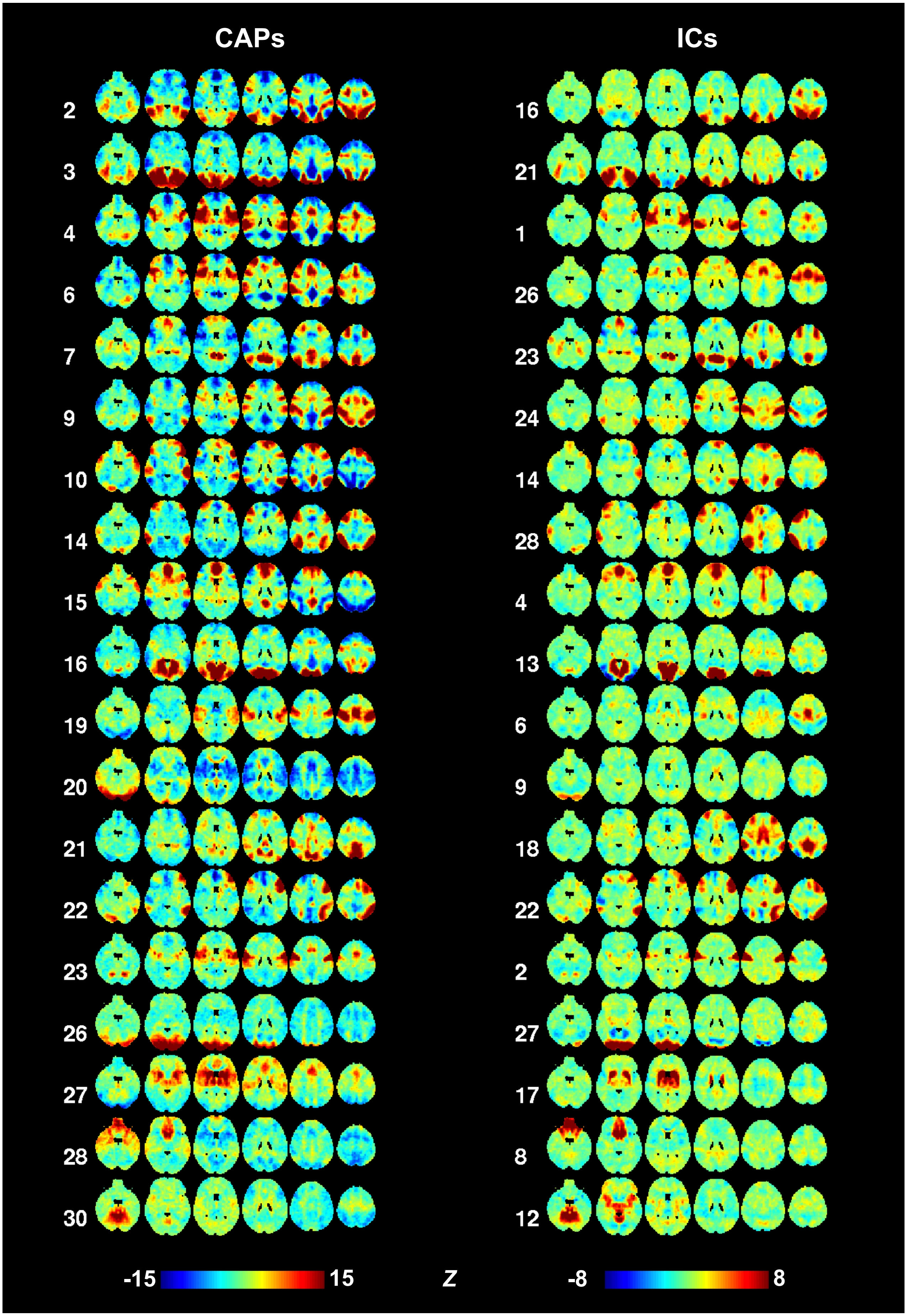

CAPs
ICs
2
3
4
6
7
9
10
14
15
16
19
20
21
22
23
26
27
28
30
16
21
1
26
23
24
14
28
4
13
6
9
18
22
2
27
17
8
12
-15
15
Z
-8
8

Fig. S7. 19 matched CAP−IC pairs, defined as those having highest spatial correlation to each other among their own categories. Although some of the CAPs show a coarse similarity, close inspection revealed distinct differences. For example, CAP 23 and IC 2 both cover sensorimotor areas, but the co-activation in supplementary motor area (SMA) is strong in CAP 23 but absent in IC 2. In addition, many CAPs show strong negative statistics indicating de-activations in a wide range of regions, while negative statistics in the ICs are generally much weaker.

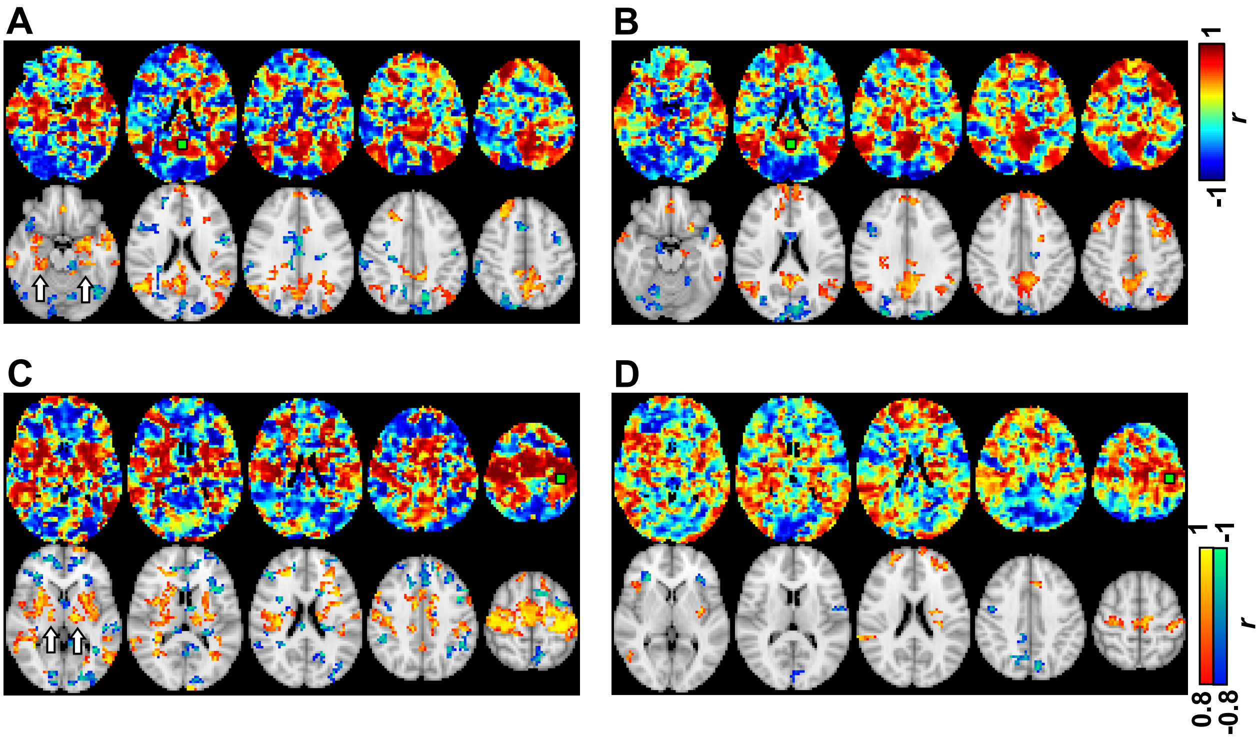

Fig. S8. PCC-seeded correlation maps from a representative subject (the same one in Figure 1) using a short sliding window (16.1-sec, 7 time points). The sliding window was centered at T11 (A), T12 (B), T3 (C), and T9 (D) shown in Figure 1, respectively. Each correlation map is shown without (upper row) and with (lower row) being overlaid on corresponding anatomical images.

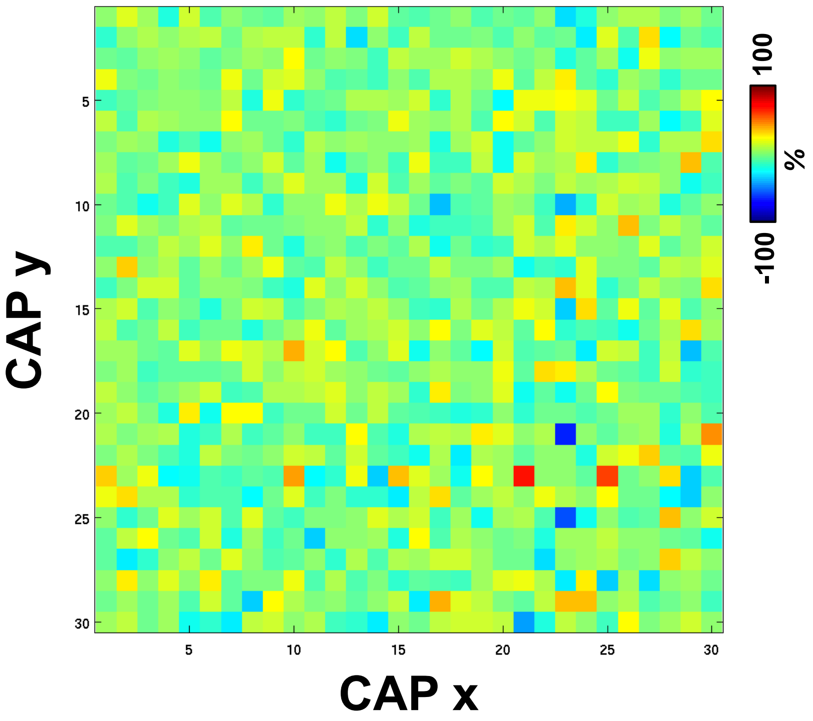

Fig. S9. Symmetric temporal relationship of CAPs. The matrix shows the difference between probabilities of seeing CAP x ahead of CAP y (within 2 TR) and of seeing CAP x behind CAP y (within 2 TR). The small values suggest a symmetric appearance of x-y and y-x sequences. We further tested whether such differences are significant under the null hypothesis that we have equal probability of seeing either of them first. The testing based on a binomial distribution model found none of these differences significant at level of 0.05 after Bonferroni correction, even for the CAP 23 – CAP 25 and CAP 21 – CAP 23 pairs that showed the highest values in the matrix.

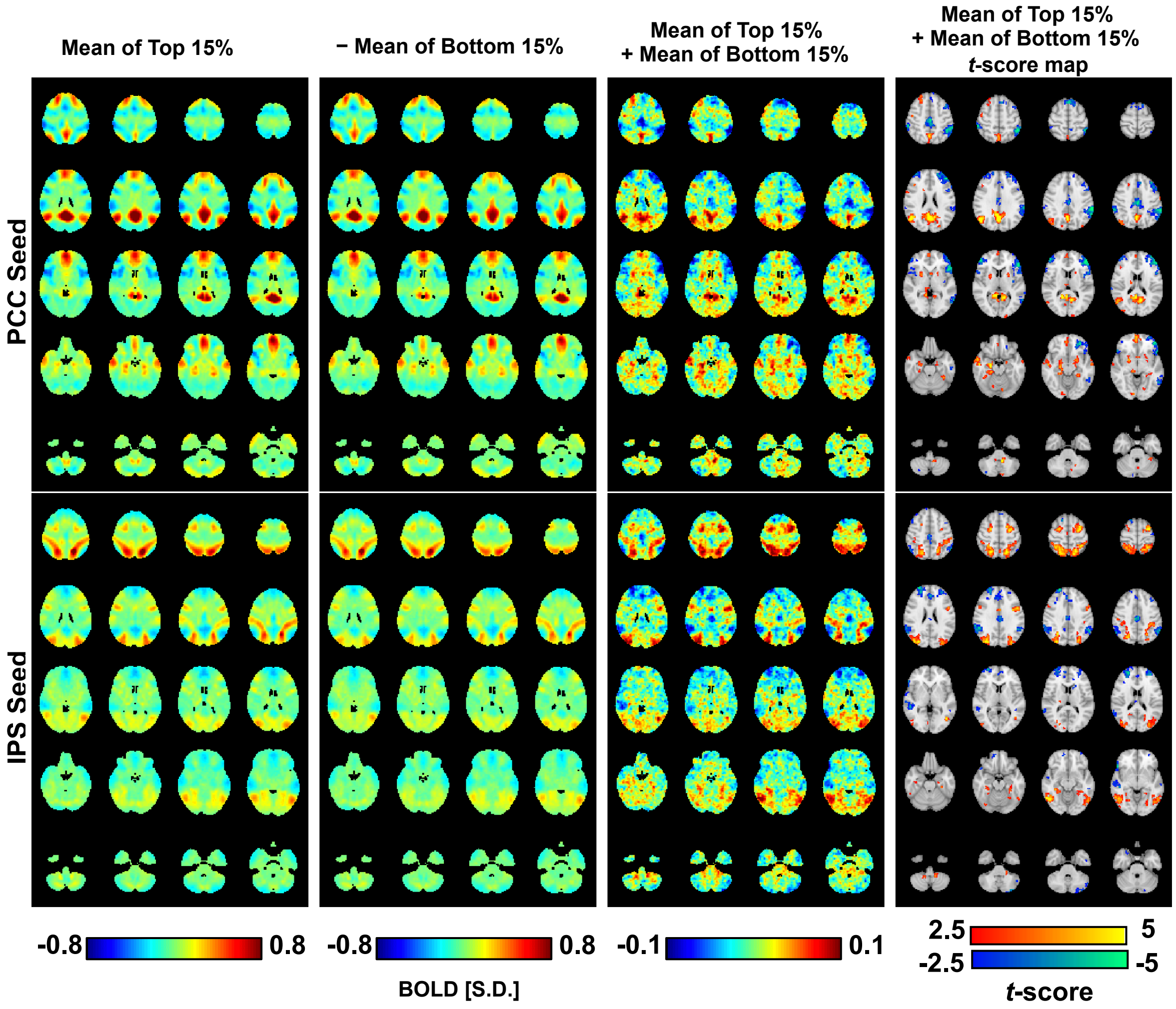

Fig. S10. A comparison of co-activation and co-deactivation patterns using 15% of the data at top and bottom ranges. The first column is the mean image of the top 15% time frames selected based on corresponding seeds (the posterior cingulate cortex, PCC; and the Intraparietal sulcus, IPS) while the second column is the mean image of the bottom 15% frames with its sign flipped; the third column is the sum of the two mean images, corresponding to the difference between the left and middle column. Although by flipping the sign, the co-deactivation shows a similar pattern as the co-activation, there is a clear difference (10~15%, note the scale in color bar) between them with distinct spatial patterns. The difference tends to be positive within seed-related regions (“task-negative” regions for PCC and “task-positive” regions for IPS) but negative in the negatively correlated regions.
